# Concordant Biogeographic Patterns among Multiple Taxonomic Groups in the Mexican Freshwater Biota

**DOI:** 10.1371/journal.pone.0105510

**Published:** 2014-08-19

**Authors:** Benjamín Quiroz-Martínez, Fernando Álvarez, Héctor Espinosa, Guillermo Salgado-Maldonado

**Affiliations:** 1 Universidad Nacional Autónoma de México, Instituto de Biología, Laboratorio de Helmintología, México D. F., México; 2 Universidad Nacional Autónoma de México, Instituto de Biología, Colección Nacional de Crustáceos, México D. F., México; 3 Universidad Nacional Autónoma de México, Instituto de Biología, Colección Nacional de Peces, México D.F., México; Consiglio Nazionale delle Ricerche (CNR), Italy

## Abstract

In this paper we analyse the degree of concordance in species richness and taxonomic distinctness (diversity) patterns among different freshwater taxonomic groups in order to test three long held patterns described in Mexican freshwater biogeography: 1. The aquatic biota of Mexico includes two distinct faunas, a rich Neotropical component in the south and a south-eastern region and a less rich Nearctic component towards central and northern latitudes of the country. 2. A hotspot of species richness and diversity has been recorded in the Usumacinta, including the Yucatan Peninsula. 3. The presence of two distinct biotas in Mexico, an eastern one distributed along the Gulf of Mexico slope, and a western one associated to the Pacific versant. We use species richness and taxonomic distinctness to explore patterns of diversity and how these patterns change between zoogeographical regions. This paper points out a clear separation between Neotropical and Nearctic drainage basins but also between eastern (Gulf of Mexico) and western (Pacific) drainage basins. Present data gives additional empirical support from freshwater biota for three long held beliefs regarding distributional patterns of the Mexican biota. The neotropical basins of Mexico are generally host to a richest and more diversified fauna, that includes more families, genera and species, compared to the less rich and less diverse fauna in the nearctic basins.

## Introduction

Mexico is recognised as one of the top five megadiverse countries [1]. Among the causes of this high biodiversity are: Mexico’s geographical position that overlaps, between two oceans (Pacific and Atlantic), tropical and subtropical areas and two biogeographical realms (Nearctic and Neotropical) [2], [3], [4], [5]; a highly variable geographic and physiographic setting resulting from a geological history characterised by intense tectonic activity and periods of marine incursions [3], [6], [7]. Several biogeographic generalizations about the distribution of the Mexican biota, have been described but most of these have resulted from analyses of individual groups, mainly from terrestrial flora or fauna [8], [9], [10], [11], [12].

The study of concordance of distributional patterns amongst different biological groups can help determine the factors involved in shaping these patterns; for example suggesting that different groups are responding to similar environmental gradients across different spatial scales or what historical factors have contributed to present-day distributional patterns [13]. In this way the combined analysis of different taxa allows a more robust delimitation of biogeographical boundaries and distributional patterns. In addition, concordance between different groups also has been analyzed as a tool for the characterisation of biodiversity surrogates or indicators [14]. Most empirical support for concordance patterns comes from terrestrial ecosystems and biota and the freshwater studies that have tackled this issue come mainly from northern temperate latitudes [13], [15], [16], [17], [18]. The degree of concordance of biogeographic patterns for different taxonomic groups of freshwater fauna has been seldom examined in neotropical regions [14], [19], [20], [21] although Tisseuil et al. [22] included neotropical regions in their analysis of spatial concordance in global diversity patterns for five freshwater taxa. Also, few studies have focused on this issue using the Mexican biota, and to our knowledge, only Huidobro et al. [23] have undertaken the study of concordance among distributional patterns of Mexican freshwater groups. More studies of this kind are needed from tropical latitudes to at least objectively verify the generality of these patterns.

In this study we examined the degree of concordance in species richness and taxonomic distinctness (diversity) patterns among different freshwater taxonomic groups in order to test three long held patterns described in Mexican freshwater biogeography: 1. The aquatic biota of Mexico includes two distinct faunas, a rich Neotropical component in the south and a south-eastern region and a less rich Nearctic component towards central and northern latitudes of the country [23], [24], [25], [26], [27]. 2. A hotspot of species richness and diversity has been recorded in the Usumacinta province (sensu Bussing [28] as updated by Matamoros et al. [29]), including the Yucatan Peninsula. 3. The presence of two distinct biotas in Mexico, an eastern one distributed along the Gulf of Mexico slope, and a western one associated to the Pacific versant [8], [9], [11], [12].

In order to explore these patterns, we examined a database that includes fishes (Poecilidae), crustaceans (Palaemonidae and Pseudothelphusidae) and helminth parasites of freshwater fishes (Nematoda, Acanthocephala, and Platyhelminthes, including Trematodes, Monogeneans, and Cestodes) in 22 river basins from Mexico. In doing so, we examine the concordance in distributional patterns amongst these Mexican freshwater groups.

## Materials and Methods

**Figure 1:**
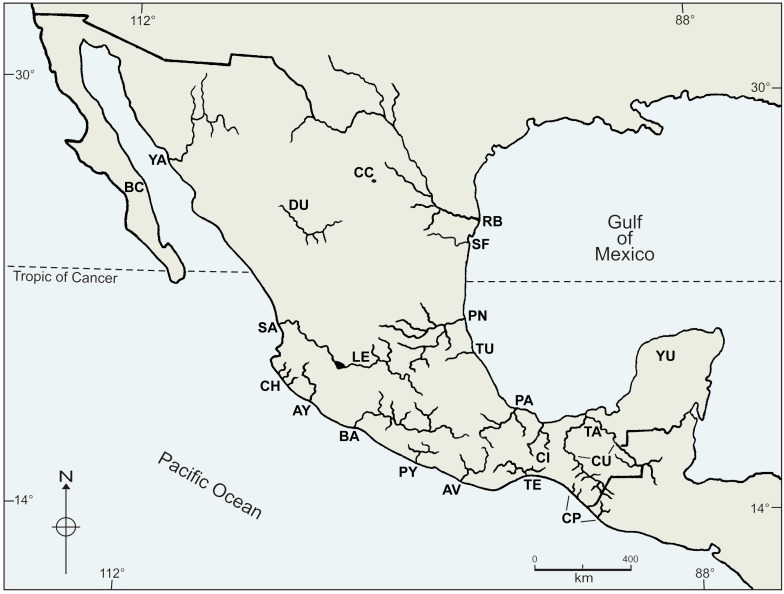
Mexican hydrological features. The code used to identify each basin is: BC, Oases of Baja California Sur; YA, Río Yaqui; CH, rivers near Chamela, Jalisco; SA, Río Santiago; AY, Río Armería-Ayuquila; BA, Río Balsas; PY, bodies of water in Guerrero, including Río Papagayo; AV, Río Atoyac-Verde; TE, Río Tehuantepec; CP, rivers along the south Pacific coast of Chiapas; RB, Río Bravo; LE, Río Lerma; CC, bodies of water of the Valley of Cuatro Ciénegas; DU, Río Mezquital, Río Nazas and springs of Durango; SF, Río San Fernando, Río Soto La Marina and other bodies of water in Tamaulipas; PN, Río Pánuco; TU, Río Tuxpan; CI, bodies of water of Los Chimalapas; PA, Río Papaloapan; TA, bodies of water in coastal plain of Tabasco; CU, basins of Río Usumacinta, Chiapas; YU, bodies of water of the Yucatán Península.

We revisited and updated the databases already published by Huidobro et al. [23] and Salgado-Maldonado & Quiroz-Martínez [27]. This updated presence/absence database includes recent data from biological surveys performed during the last decade and represents each species of helminth parasites of freshwater fishes (Nematoda, Acanthocephala, and Platyhelminthes, including Trematodes, Monogeneans, and Cestodes), crustaceans (Palaemonidae and Pseudothelphusidae) and freshwater fish (Poecilidae) found in each of the 22 hydrological basins used in this study (Table S1). These groups are representative of inland freshwater biota of Mexico, are widely distributed and have endemic genera and species. Particularly, the family Poeciliidae is a widespread and diverse group, endemic to the New World with majority of the species occurring in Mexico, Central America and the Antilles [30]. Location of the basins and the code used to identify each one in the subsequent text and plots are shown in Fig. 1. The information included in the initial matrix was subsequently aggregated into the corresponding supra-generic levels, such that for every species it includes the relationships to genus, family, class, and phylum, updated from the previous taxonomical scheme available from Salgado-Maldonado [31], Espinosa-Perez [32] and Álvarez et al. [33].

The Average taxonomic distinctness (Δ^+^) was calculated using the next function:
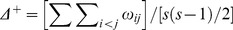
(1)where ω*ij* is the taxonomic path length between species *i* and *j*, and *s* is the number of species. The average taxonomic distinctness (Δ^+^) measures the average taxonomic distance between different species in an assemblage; the greater the value of Δ^+^, the greater the average taxonomic difference between species in the assemblage [34]. The computation of the index follows the taxonomic hierarchy based on the Linnaean classification into phyla, classes, families, genera and species; it was made using the Plymouth Routines in Multivariate Ecological Research PRIMER v6 [35], [36].

Using the inventory of freshwater species recorded in Mexico [27], constructed from individual lists of species recorded in each drainage basin, we identified differences in taxonomic distinctness (Δ^+^), from expected Δ^+^ values derived from the total species list. We performed a randomization procedure (as suggested by Clarke & Warwick [37], [38] and by Warwick & Clarke [39]) for any observed set of species for Mexican river basins. A simulated distribution was developed leading to a theoretical mean (the horizontal line shown in the graph of Δ^+^ for the 22 basins against richness in each basin) and to a confidence funnel for each, Δ^+^, from random subsamples as suggested by Bhat and Magurran [40]. Values of Δ^+^ located within the 95% probability funnel indicate that species diversity in the corresponding areas falls within the expected range, thus allowing for both, sample size and sample effort free, diversity comparisons.

In addition, we calculated two indices to compare the similarity/dissimilarity between the various basins: 1) Sørensen’s compositional similarity index [41], and 2) the taxonomic dissimilarity index (θ^+^), as defined by Warwick & Clarke [42] and Clarke & Warwick [38]. The taxonomic dissimilarity index, which is a presence/absence-based ‘‘beta-diversity’’ coefficient [36], is a natural extension of the index of taxonomic distinctness Δ^+^ [38]. The resulting matrices were examined to derive dissimilarity patterns by means of both cluster analysis (group average linkage) and non-metric multidimensional scaling (nMDS), as suggested by Field et al. [43] and Clarke & Warwick [36] using Matlab software.

## Results

**Figure 2:**
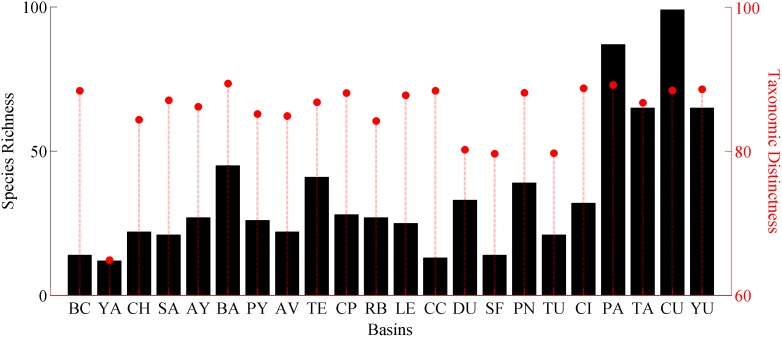
Patterns of richness (bars) and diversity, taxonomic distinctness measure, Δ+ (markers) for 22 freshwater basins of Mexico (codes for basins the same that in map, Fig. 1).

**Figure 3:**
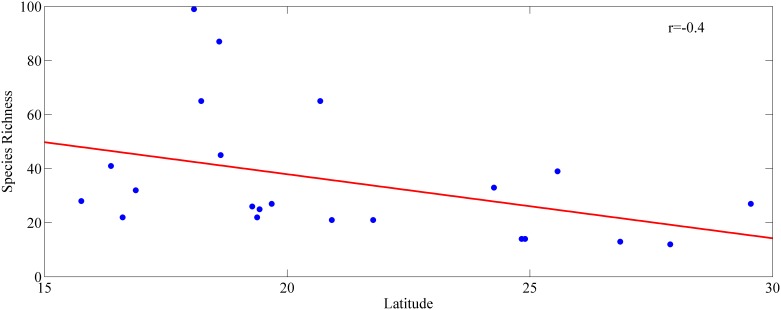
Relationship between species richness and latitude for all samples.

Our revisited database includes a total of 332 species from 84 genera and 34 families belonging to five different *phyla* (Platyhelminthes, Acanthocephala, Nematoda, Arthropoda and Chordata), recorded from 22 drainage basins across Mexico (Fig. 1). Species richness varied widely throughout drainage basins in the country (Fig. 2). However, the variation in species richness characterises Neotropical and Nearctic basins because Neotropical basins from south and south-east Mexico are generally higher in species richness (S = 68–99). While the Nearctic basins in northern and central Mexico, north of the Trans-Mexican Volcanic Belt (≈19° latitude) have lower species richness (S = 12–14). Our results corroborate a species richness gradient that goes from the south-eastern basins of Mexico (Yucatan (YU), Tabasco (TA), Papaloapan (PA), and Chiapas Usumacinta (CU) (S = 68–99)), towards the less rich basins in northern Mexico (Yaqui (YA), Cuatro Cienegas (CC), Oases of Baja California (BC), San Fernando (SF) and Santiago (SA) (S = 12–14)). The Chiapas Usumacinta (CU) (total number of species counted S = 99) and Papaloapan (PA) (S = 87) river basins, plus those grouped under Tabasco (TA) (S = 68) and Yucatan (YU) basins were the richest, followed by Balsas (BA) (S = 45), Tehuantepec (TE) (S = 41) and Panuco (PN) (S = 39) river basins. Contrarily, the observed richness for the rest of the studied basins ranged from S = 12 to S = 33 (Figs. 1, 2). Correlation between species richness and latitude was negative but not statistically significant (Fig. 3, r = −0.4, p>0.05).

**Figure 4:**
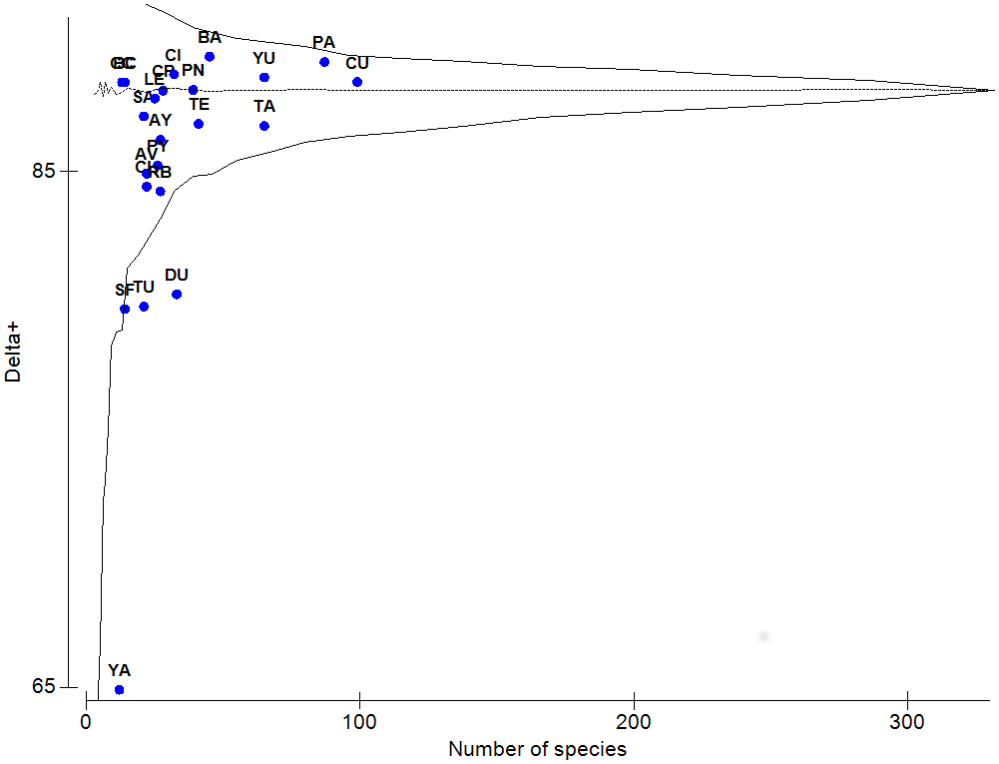
Simulated distribution of average taxonomic distinctness measure, Δ+, (theoretical mean, horizontal, dashed line) for random subsets of 322 species from 22 drainage basins of Mexico, and the 95% confidence limits (funnel) of taxonomic distinctness. Superimposed to the theoretical model are shown the actual values of diversity, Δ+, for each of the 22 Mexican basins.

Diversity, measured as taxonomic distinctness, Δ^+^, is more evenly distributed among basins; however it also allows for the differentiation of Neotropical and Nearctic basins (Fig. 2). Most of the Δ^+^ values of diversity were close to those expected under the simulation funnel (Fig. 4) indicating that most of the Mexican basins are as diverse as expected. However, the San Fernando (SF), Tuxpan (TU), Durango (DU) and Yaqui (YA) river basins are below the lower limit of the simulated distribution, a result that reflects a historical low sampling effort in these areas. Diversity among basins does not follow the same pattern of variation as richness; Δ^+^ diversity values and observed richness, S, were not correlated (*r* = 0.48). The higher values of Δ^+^ diversity (Figs. 2, 4) were recorded in the Balsas (BA), Papaloapan (PA) and Chimalapas (CI) basins; although not the richest in terms of species numbers, these three basins have higher numbers of taxonomic categories, their records include all higher taxa (Acanthocephala, Platyhelminthes, Nematoda, Arthropoda and Chordata), with a noticeable evenness in the distribution of genera and species in classes and phyla. High values of Δ^+^ were also recorded for the Yucatan (YU), Chiapas Usumacinta (CU), Baja California (BC), Cuatro Cienegas (CC), Panuco (PN), and Chiapas Pacifico (CP) basins. Comparatively, lower values of Δ^+^ were recorded for Lerma (LE), Santiago (SA), Tehuantepec (TE), Tabasco (TA), Ayuquila (AY), Papagayo (PY), Atoyac-Verde (AV), Chamela (CH), and Rio Bravo (RB) basins; the lowest values for diversity, Δ^+^, were recorded from Durango, Tuxpan, San Fernando, and Río Yaqui (Figs. 2, 4) basins where all five phyla and classes have been recorded; however, the distribution from lower to higher taxa is less even, with a clear dominance by certain groups.

Regarding only species richness, having Chiapas Usumacinta (CU), Papaloapan (PA), Tabasco (TA) and Yucatan (YU) as the richest basins (S = 65–99) points out the Usumacinta Province as a hotspot. However, concerning Δ^+^ diversity the Balsas (BA) river basin had the highest values; followed by Chimalapas and all the basins previously mentioned as the Usumacinta province, excepting Tabasco (TA). Unexpectedly, Tabasco (TA) lies outside the nucleus of most diverse basins. As a consequence, the distinction of a hotspot of richness and diversity located in the Usumacinta province from this Δ^+^ diversity approach is less clear. Moreover, Balsas (BA), Baja California (BC), Cuatro Cienegas (CC) and Chiapas Pacifico (CP) arise as basins with a noticeable high Δ^+^ diversity.

**Figure 5:**
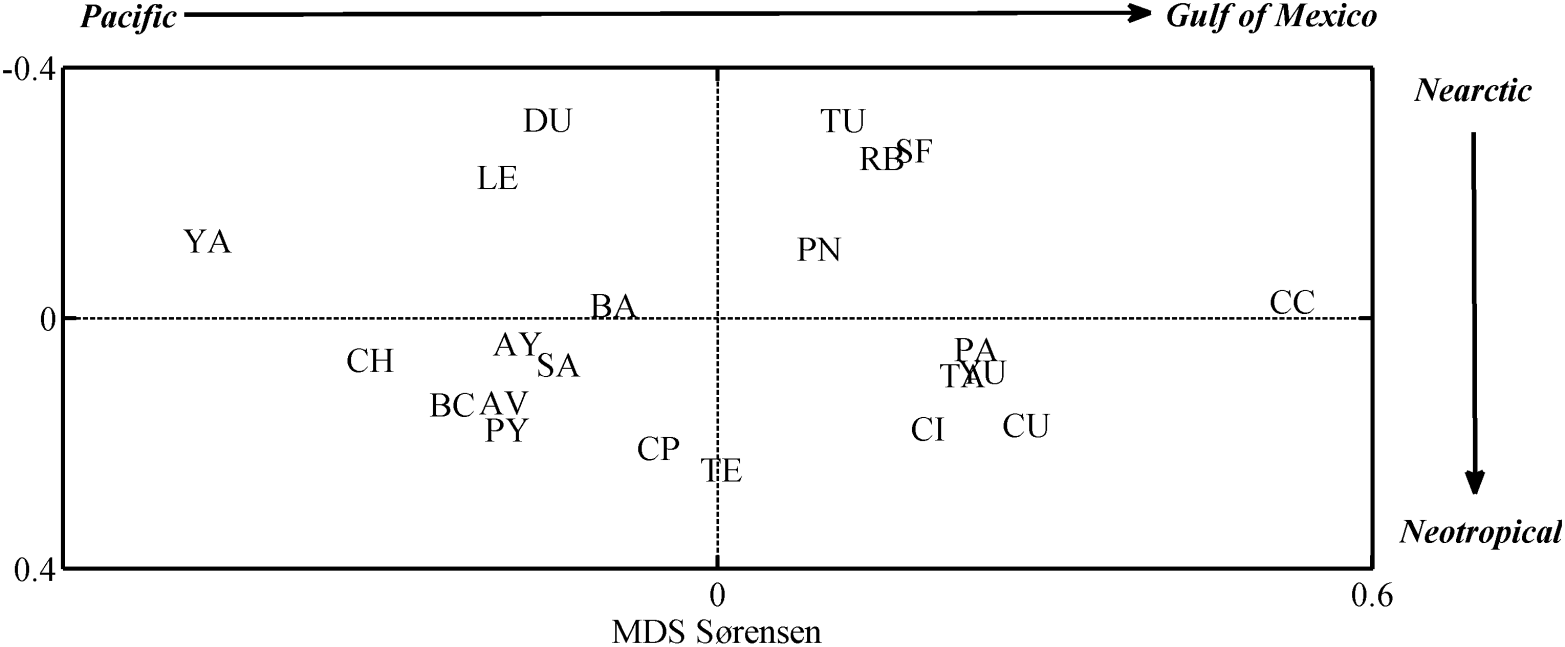
Non metric multidimensional scaling nmMDS, ordination plot resulting from similarity matrix based on Sorensen’s index values for 22 Mexican hydrological basins.

**Figure 6:**
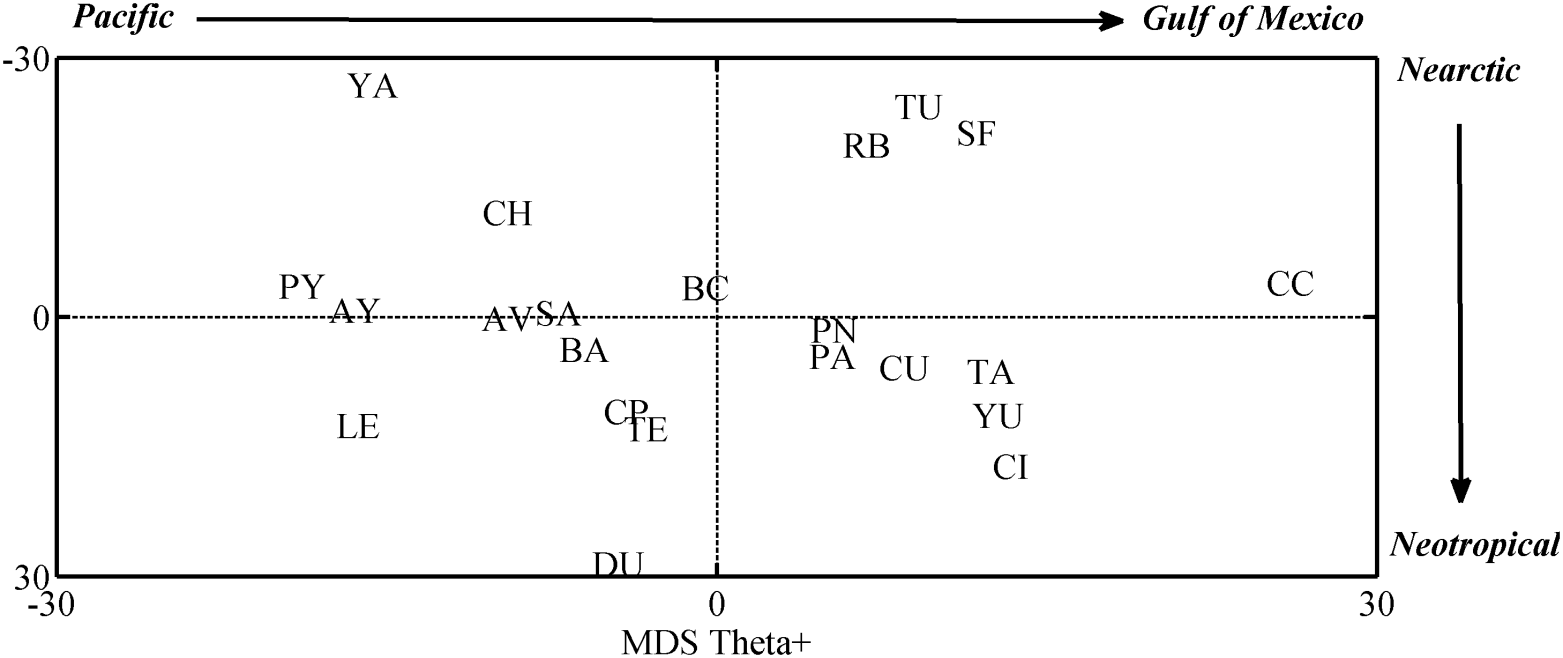
Non metric multidimensional scaling nmMDS, ordination plot resulting from dissimilarity matrix based on taxonomic distinctness, Δ+, values for 22 Mexican hydrological basins.

Analysis of similarity, based on the Sørensen’s coefficient and on taxonomic distinctness index (θ^+^), between freshwater faunas of the 22 drainage basins for all species stresses the existence of a Pacific-Gulf of Mexico (east-west) divide and also a Nearctic-Neotropical (north-south) divide. Figures 5 and 6 represent the resulting MDS ordinations which show: 1) A large suite of Gulf of Mexico (eastern) species divided into two subgroups; one composed mainly of fauna recorded from Neotropical basins such as Yucatan (YU), Tabasco (TA), Papaloapan (PA), Chimalapas (CI) and Chiapas Usumacinta (CU) river basins and another composed by faunas from Nearctic basins such as Panuco (PN), Tuxpan (TU), San Fernando (SF) and Rio Bravo (RB) basins. 2) A second group of Pacific (western) species also divided into two subgroups; one Neotropical that includes the Tehuantepec (TE), Chiapas Pacifico (CP), and Papagayo (PY) river basins the Rio Lerma (LE) and the bodies of water from Durango (DU) and one Nearctic including the fauna from the Balsas (BA), Santiago (SA), Ayuquila (AY), Atoyac-Verde (AV), Baja California (BC) and Chamela (CH) basins (Figs. 5, 6).

The analysis of each group separately showed that fishes and crustaceans follow the described patterns very closely; however the distribution of helminth parasites of freshwater fishes only shows partial concordance with these patterns. The north-south gradient is followed by all five phyla but the west-east patterns in helminth parasites are not as clear as they are for fishes and crustaceans (Figs. S1, S2 and S3).

## Discussion

Our analyses showed a concordance in distributional patterns of freshwater fauna including helminth parasites of freshwater fishes, crustaceans and fishes supporting the biogeographical division of Mexico along a north-south axis. We have also confirmed that the drainage basins of southeastern Mexico harbour a richer, predominantly Neotropical fauna, while, in general, the basins of the Mexican Highland Plateau and the Nearctic area of Mexico harbour a less diverse temperate fauna. An area of high diversity can be distinguished in the Usumacinta province; however, the presence of a hotspot in this province is nuanced by the low values of Δ+ found for Tabasco (TA). The lower values of diversity found in Tabasco are probably a consequence of: crustaceans being exclusively represented by five widely distributed species of the freshwater prawn genus *Macrobrachium*, the absence of species of the fish genus *Poeciliposis*, and the presence of several genera of helminths with three or more species such as *Sciadicleithrum*. We also found unexpected areas of high diversity (Δ^+^) in other regions such as the Balsas (BA), Baja California (BC), Cuatro Cienegas (CC), Panuco (PN) and Chiapas Pacifico (CP) basins. The Oases of Baja California although poor in species richness (S = 14) included all five higher taxa (phyla Platyhelminthes, Acanthocephala, Nematoda, Arthropoda and Chordata) with only Pseudothelphusid crabs missing from the records. Similarly, Cuatro Cienegas shows low species richness (S = 13) but its records included four of the five higher taxa (phyla Platyhelminthes, Nematoda, Arthropoda and Chordata). In addition, two species of Poecilidae and one Palaemonidae are endemic to Cuatro Cienegas, there are however, to date no endem­ic species of helminth recorded in this area [27]. Cuatro Cienegas is a transitional zone of neotropical and temperate climate zones, and so is a mixing point where different species may co-occur [44]. On the other hand, the Panuco basin’s complex topography favours the development of a diversity of ecosystems as well as a diverse biota [45], however we found moderate species richness (S = 39) although the records include all five higher taxa (phyla Platyhelminthes, Acanthocephala, Nematoda, Arthropoda and Chordata).

The concordance in distributional patterns of the groups of freshwater fauna examined in this study gives additional support to the long held pattern of the Nearctic-Neotropical divide of the Mexican biota. Our data shows this divide not only based on species richness but also from a sound evaluation of diversity by means of the taxonomic distinctness index (Δ^+^). More species and more taxa are recorded in Neotropical basins, also these basins are characterised by a more even distribution of higher level taxa. In contrast, Nearctic basins are less rich and display a less even distribution of taxa. We acknowledge that an asymmetrical sampling could have an impact on our results; the Mexican tropical river basins have been studied far more intensively, with a greater number of basins explored and more frequency in sampling. Still, our work suggests that the pattern of increased richness in tropical environments is true in the case of helminth parasites, fishes and crustaceans of Mexico. The faunal complexity of south-eastern Mexico’s hydrological basins is much larger than the basins of central Mexico; for example, the Usumacinta and Grijalva basins harbour 111 species of fish and 51 of helminth parasites, while the Lerma basin is inhabited by 52 species of fish and 20 species of helminths.

The results of the cluster analysis divide the country in an east-west axis; this is consistent with the findings of Morrone and Marquez [12], Escalante et al. [8], [9] and Morrone [11], who distinguished an east to west biotic divide in Mexico. Our analyses provide additional empirical support to the patterns described by these authors for terrestrial biota. This east–west divide does not contradict the classical north–south axis that roughly divides Mexico into northern and southern portions on both sides of the Trans Mexican Volcanic Belt; it also helps explain Mexican biotic complexity [9], [46], [47], [48]. This pattern corresponds to the actual orographic configuration of the country considering that the main mountain ranges constitute obstacles for invading inland areas of Mexico and that the Neotropical groups mainly originated in Central and South America, or even southern Mexico (poecilids [30], cichlids [49]). This pattern of dispersion can be explained by an invasion of aquatic biota during the Paleocene thus giving additional empirical support to Escalante et al. [9]. The division between the Nearctic and Neotropical regions incorporates the two biotic divisions, the north–south Miocene axis and the east–west Paleocene line [9], [50], [51].

Our data also give additional empirical support to the subregions in the Mexican Neotropical region recognised by Escalante et al. [8]. The subregions proposed were Pacific-Central America, Mexican Gulf-Central America, and Central America. The first one includes the Pacific coast from Sinaloa, Mexico, southwards to Central America. The second one includes provinces mainly in the lowlands of the Yucatan peninsula, Mexican Gulf-coast, and Central America.

The patterns herein described also complement the findings by Huidobro et al. [23] where the presence of two distinct faunas distributed along both Mexican coasts, stemming from a bifurcation in the Isthmus of Tehuantepec, is suggested by the generalized tracks proposed by these authors. This is consistent with the present day physiography of Mexico, where the two large mountain ranges, the Sierra Madre Occidental and the Sierra Madre Oriental, induce rivers to drain either to the east toward the Gulf of Mexico, or to the west toward the Pacific Ocean, and so determining dispersal routes for freshwater biota along the outer margins of these mountain ranges [27].

As shown by analyses of diversity, our results provide additional evidence to consider the Chimalapas and Tehuantepec basins as sites of high freshwater diversity. The Isthmus of Tehuantepec represents a node of species diversity and is an important region for dispersal across Mexico; it is considered a bifurcation zone, that has directed the neotropical lowland fauna towards coastal environments, with one branch extending towards Oaxaca and the other one towards Veracruz and Tabasco [23], [52]. Rodriguez and Magalhães [53] stressed a maximum concentration of genera and species in the neighbouring areas east of the Isthmus of Tehuantepec. Nearly traversed by the Coatzacoalcos River, this low-altitude, narrow isthmus is the only region in Mexico where multiple groups of aquatic and riparian animals appear to have spread between the Gulf of Mexico and Pacific drainages [52], [54], [55].

The distribution of helminth parasites of freshwater fishes reflects the same pattern described above as mentioned by Vidal-Martínez and Kennedy [56] and Aguilar-Aguilar and Salgado-Maldonado [2], [57]. In recent studies, Quiroz-Martínez and Salgado-Maldonado [25], [26] were able to discriminate in addition to the Neotropical and Nearctic groups, a group with Pacific affinity.

Álvarez and Villalobos [24] showed that the Mexican Chiapas State is an area of high diversification for pseudothelphusids. Seven genera and 13 species, representing three of the five tribes that compose the subfamily Pseudothelphusinae are found in Chiapas. The distribution of the tribe Pseudothelphusini corresponds to a strict Neotropical pattern, extending throughout south-central Mexico and the Pacific slope, and reaches the southern part of Sonora; this represents the northernmost limit of the entire Pseudothelphusidae family. They are, however, absent from the Yucatan Peninsula, northern Veracruz, and the rest of the northern Gulf of Mexico slope [58].

A total of 22 species of *Macrobrachium* have been recorded from Mexico; seven of them are distributed on the Pacific slope only, 13 occur along the Gulf of Mexico slope, and two occur on both versants [59], [60], [61], [62], [63], [64], [65], [66]. Of the 13 species from the Gulf of Mexico, nine have abbreviated development, occur in the upper reaches of basins, have rather reduced geographic ranges and all of them occur in only one basin.

The same pattern holds for poecilid fishes as the distribution of the genus *Poeciliopsis* is primarily restricted to Pacific slope drainages of Mexico and is notoriously absent from the Gulf of Mexico drainages north of the Trans-Mexican Volcanic Belt [52], whereas *Poecilia* is found mainly along the Atlantic (Gulf of Mexico) slope drainage basins [45]. However, they are a conspicuous faunal component of Central America, accounting for approximately 35% of the secondary freshwater fauna [67].

Similarities in species composition and distribution of richness among hydrological basins are consistent with the notion of the Usumacinta Ichthyological Province restricted to the northern part of Central America, including Yucatan, as proposed by Miller [45] and updated by Smith and Bermingham [68] and Matamoros et al. [29], [69]. Our results give additional empiric support to the work of Matamoros et al. [29] in that we characterised a hotspot of richness and diversity restricted to the Usumacinta province including the Yucatan Peninsula. Moreover, Tabasco along with the Papaloapan River basins are considered by Morrone [10] part of the Gulf of Mexico biogeographical province, while Yucatan is considered as a fairly independent province [10].

There is not, however, a complete concordance between the patterns of distribution of helminth parasites and those found for fishes and crustaceans. East-west patterns in helminth parasites are not as clear as they are for fishes and crustaceans. According to several authors, this could be interpreted by a delayed cospeciation, in which parasite speciation lags behind host speciation [70], [71], [72], [73], [74]. Johnson et al. [75] and Mendoza-Franco and Vidal-Martínez [73] mentioned that host switching could explain this apparent failure from the part of parasites to speciate in response to host speciation. Assuming that morphological and physiological characters of host fish belonging to the same family should remain relatively similar, there should be no extreme barriers for helminth parasites to infect a new host species [73]. Quiroz-Martínez and Salgado-Maldonado showed that most genera of helminths are monospecifically represented, confirming that there are not many congeneric species in the helminth fauna of freshwater fishes from Mexico and Central America [26], [27]. This suggests that helminths exploit a narrow range of host species by infecting mostly fish belonging to the same family. In helminth communities mainly structured by host-switching, parasites would not track host species, but would tend to track host resources that could be represented across different host taxa [74].

Our findings provide ample evidence that the freshwater fauna can be used to characterise hydrological basins and that there is congruence in distribution patterns of fishes and crustaceans and to a large extent in helminths. We show that the basins of south-eastern Mexico harbour a predominantly Neotropical fauna whereas the river basins from the Mexican Central Plateau and the Nearctic region are home to a different set of species. Our analyses allowed the distinction of Neotropical, Nearctic but also of eastern (Gulf of Mexico) and western (Pacific) drainage basins. Present data gives additional empirical support from freshwater biota for three long held beliefs regarding distributional patterns of the Mexican biota.

Finally, our results suggest that Δ^+^ could be an appropriate tool for conservation strategies, since it allows the identification of areas where significant numbers of species, genera and higher taxa co-occur. Taxonomic distinctness takes into account the taxonomic relatedness of species, an assemblage that harbours distantly-related species from just one family. Furthermore, these indices are largely insensitive to sampling-effort and habitat type [36], [38]. In marine ecosystems, the family of taxonomic distinctness indices has been found to perform well in assessments of anthropogenic perturbations on biodiversity [42], [76], [77], [78], [79], [80], [81]. However, in freshwater ecosystems, only a few studies have examined the performance of taxonomic distinctness indices in biodiversity evaluation and environmental assessment [40], [82], [83], [84].

## Supporting Information

Figure S1 **Dendrogram resulting from dissimilarity matrix based on taxonomic distinctness, Δ+, values for Helminth Parasites of Freshwater Fishes from 22 Mexican hydrological basins.**
(TIF)

Figure S2 **Dendrogram resulting from dissimilarity matrix based on taxonomic distinctness, Δ+, values for Crustaceans from 22 Mexican hydrological basins.**
(TIF)

Figure S3 **Dendrogram resulting from dissimilarity matrix based on taxonomic distinctness, Δ+, values for Poecilids from 22 Mexican hydrological basins.**
(TIF)

Table S1 **Presence-Absence database of freshwater fauna of Mexico used in this study.**
(PDF)
