# Supplementary figures and images for: Concordant Biogeographic Patterns among Multiple Taxonomic Groups in the Mexican Freshwater Biota
Source: PLoS One. 2014 Aug 19;9(8):e105510. doi: 10.1371/journal.pone.0105510 (PMC4138176; doi:10.1371/journal.pone.0105510)

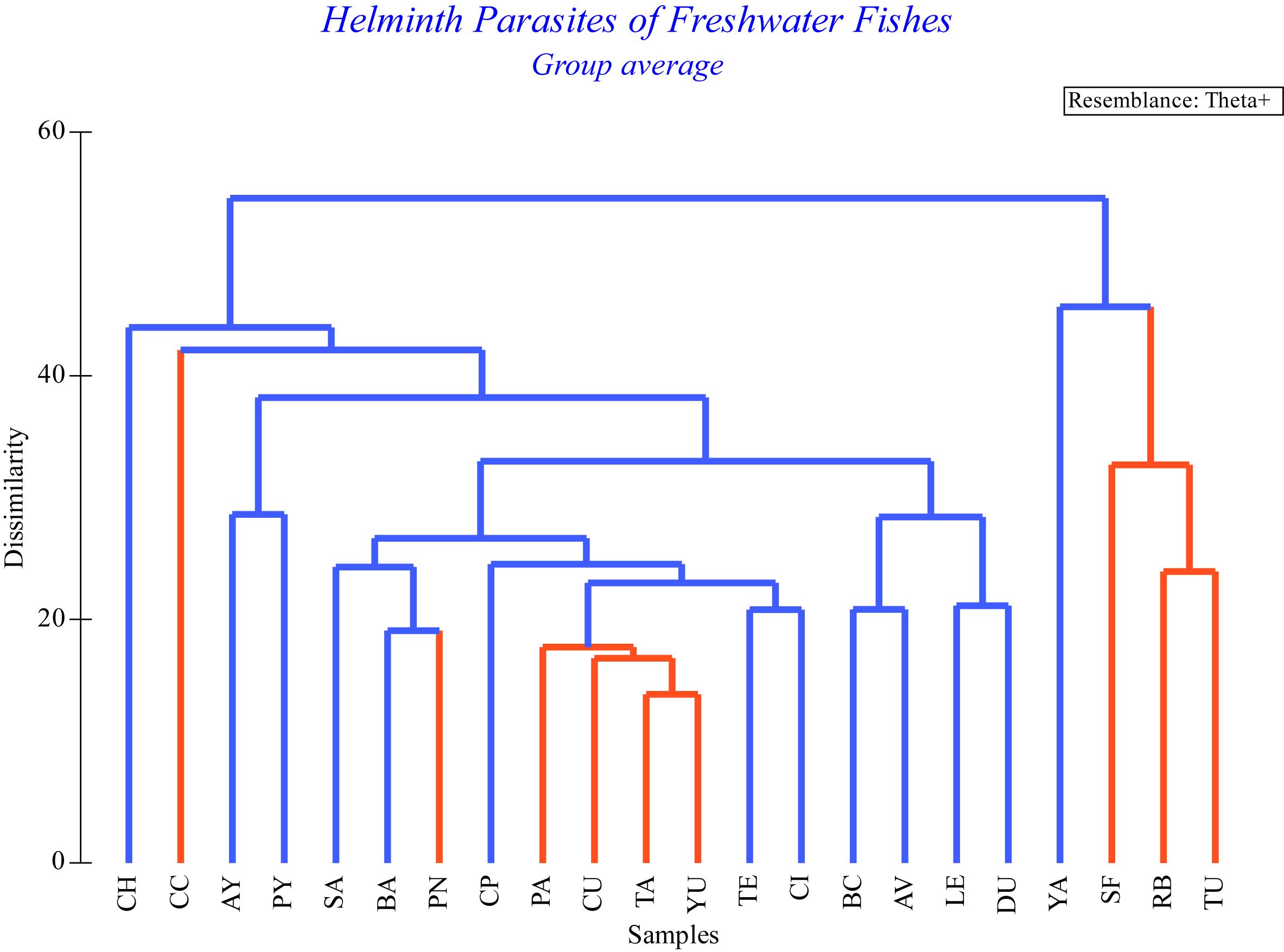

Supplement: Figure S1 — Dendrogram resulting from dissimilarity matrix based on taxonomic distinctness, Δ+, values for Helminth Parasites of Freshwater Fishes from 22 Mexican hydrological basins. (TIF) [file pone.0105510.s001.tif]

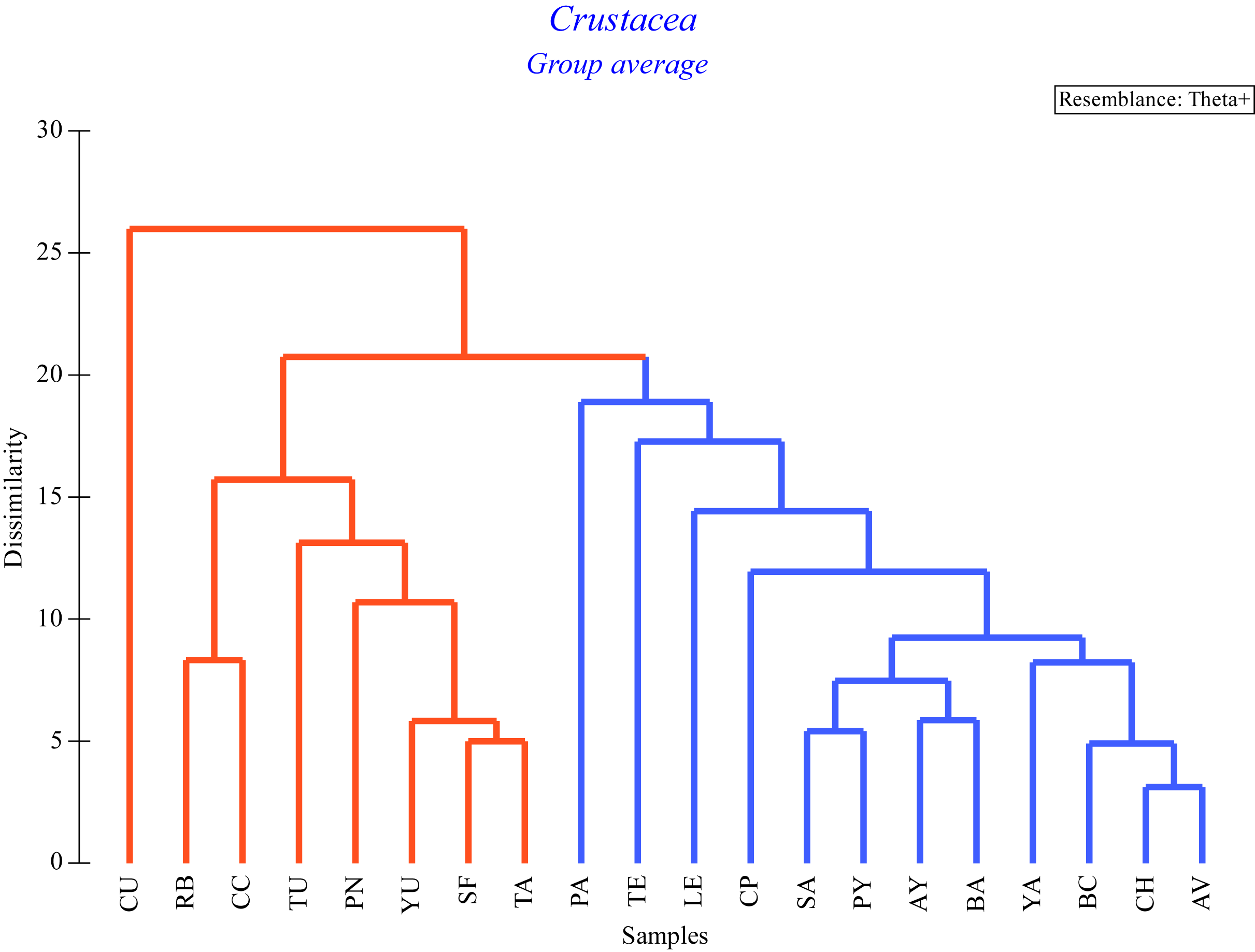

Supplement: Figure S2 — Dendrogram resulting from dissimilarity matrix based on taxonomic distinctness, Δ+, values for Crustaceans from 22 Mexican hydrological basins. (TIF) [file pone.0105510.s002.tif]

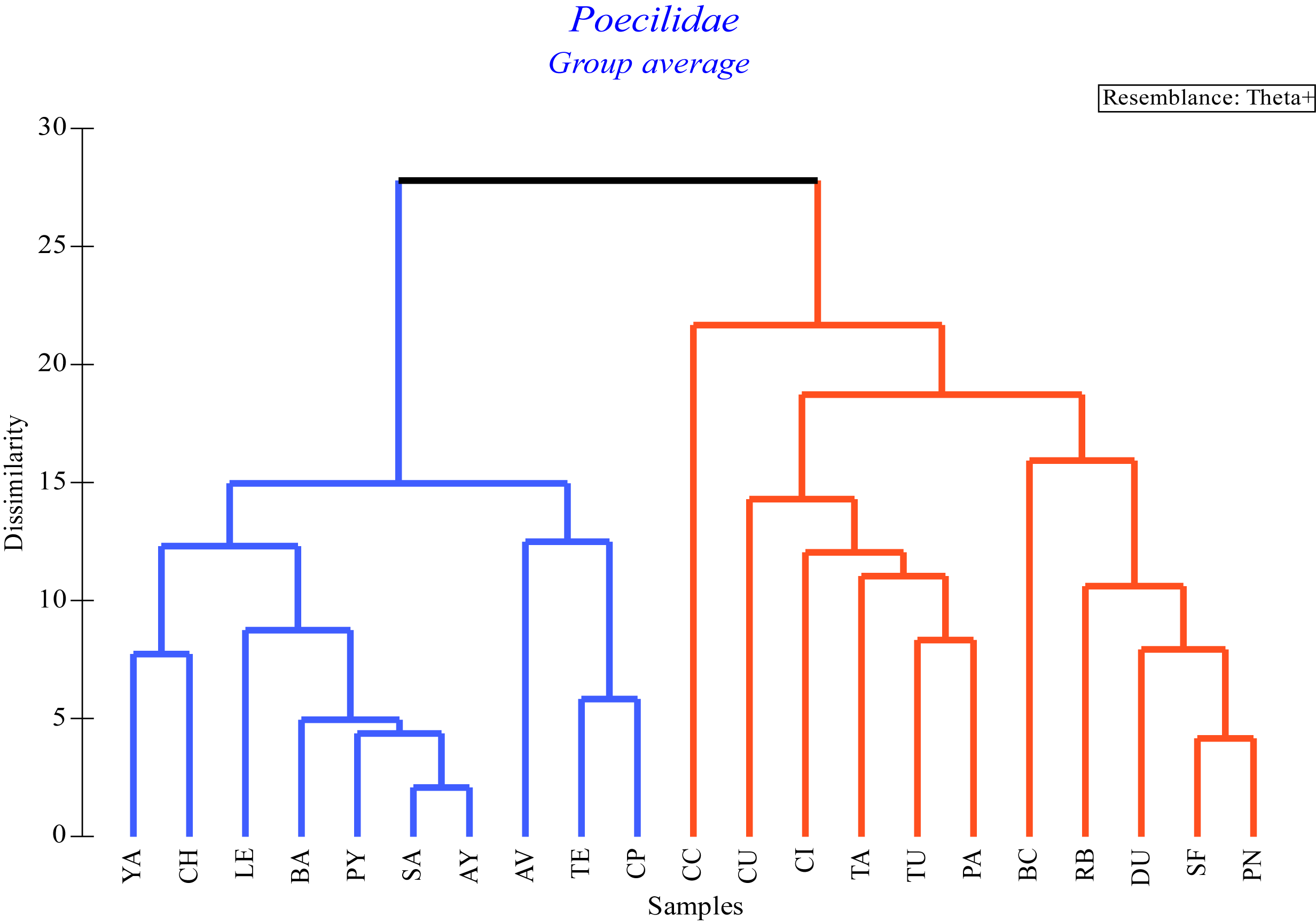

Supplement: Figure S3 — Dendrogram resulting from dissimilarity matrix based on taxonomic distinctness, Δ+, values for Poecilids from 22 Mexican hydrological basins. (TIF) [file pone.0105510.s003.tif]
