# Supplementary material for: Concordant Biogeographic Patterns among Multiple Taxonomic Groups in the Mexican Freshwater Biota
Source: PLoS One. 2014 Aug 19;9(8):e105510. doi: 10.1371/journal.pone.0105510 (PMC4138176; doi:10.1371/journal.pone.0105510)
Supplement: Table S1 — Presence-Absence database of freshwater fauna of Mexico used in this study. (PDF) [file pone.0105510.s004.pdf]

Family	Order	Class	Phylum	Species	Baja California	Río Yaqui	Chamela	Santiago	Armería	Balsas	Papagayo	Verde	Tehuantepec
Palaemonidae	Decapoda	Malacostraca	Arthropoda	*Creaseria morleyi*	0	0	0	0	0	0	0	0	0
Palaemonidae	Decapoda	Malacostraca	Arthropoda	*Cryphiops (Bithynops) luscus*	0	0	0	0	0	0	0	0	0
Palaemonidae	Decapoda	Malacostraca	Arthropoda	*Cryphiops (Bithynops) perspicax*	0	0	0	0	0	0	0	0	0
Palaemonidae	Decapoda	Malacostraca	Arthropoda	*Cryphiops (Bithynops) sbordonii*	0	0	0	0	0	0	0	0	0
Palaemonidae	Decapoda	Malacostraca	Arthropoda	*Cryphiops (Bithynops) villalobosi*	0	0	0	0	0	0	0	0	0
Palaemonidae	Decapoda	Malacostraca	Arthropoda	*Macrobrachium acanthurus*	0	0	0	0	0	0	0	0	0
Palaemonidae	Decapoda	Malacostraca	Arthropoda	*Macrobrachium acherontium*	0	0	0	0	0	0	0	0	0
Palaemonidae	Decapoda	Malacostraca	Arthropoda	*Macrobrachium americanum*	1	0	1	1	1	1	1	1	1
Palaemonidae	Decapoda	Malacostraca	Arthropoda	*Macrobrachium carcinus*	0	0	0	0	0	0	0	0	0
Palaemonidae	Decapoda	Malacostraca	Arthropoda	*Macrobrachium cosolapaense*	0	0	0	0	0	0	0	0	0
Palaemonidae	Decapoda	Malacostraca	Arthropoda	*Macrobrachium digueti*	1	1	1	1	1	1	1	1	0
Palaemonidae	Decapoda	Malacostraca	Arthropoda	*Macrobrachium heterochirus*	0	0	0	0	0	0	0	0	0
Palaemonidae	Decapoda	Malacostraca	Arthropoda	*Macrobrachium hobbsi*	0	0	1	1	0	0	1	1	1
Palaemonidae	Decapoda	Malacostraca	Arthropoda	*Macrobrachium jacatepecense*	0	0	0	0	0	0	0	0	0
Palaemonidae	Decapoda	Malacostraca	Arthropoda	*Macrobrachium mazatecum*	0	0	0	0	0	0	0	0	0
Palaemonidae	Decapoda	Malacostraca	Arthropoda	*Macrobrachium michoacanus*	0	0	1	0	1	1	0	1	1
Palaemonidae	Decapoda	Malacostraca	Arthropoda	*Macrobrachium oaxacae*	0	0	0	0	0	0	0	0	0
Palaemonidae	Decapoda	Malacostraca	Arthropoda	*Macrobrachium occidentale*	0	1	1	1	1	1	1	1	1
Palaemonidae	Decapoda	Malacostraca	Arthropoda	*Macrobrachium ohione*	0	0	0	0	0	0	0	0	0
Palaemonidae	Decapoda	Malacostraca	Arthropoda	*Macrobrachium olfersii*	1	0	1	1	1	1	1	1	0
Palaemonidae	Decapoda	Malacostraca	Arthropoda	*Macrobrachium sbordonii*	0	0	0	0	0	0	0	0	0
Palaemonidae	Decapoda	Malacostraca	Arthropoda	*Macrobrachium tenellum*	1	0	1	1	1	1	1	1	1
Palaemonidae	Decapoda	Malacostraca	Arthropoda	*Macrobrachium totonacum*	0	0	0	0	0	0	0	0	0
Palaemonidae	Decapoda	Malacostraca	Arthropoda	*Macrobrachium tuxtlaense*	0	0	0	0	0	0	0	0	0
Palaemonidae	Decapoda	Malacostraca	Arthropoda	*Macrobrachium vicconi*	0	0	0	0	0	0	0	0	0
Palaemonidae	Decapoda	Malacostraca	Arthropoda	*Macrobrachium villalobosi*	0	0	0	0	0	0	0	0	0
Palaemonidae	Decapoda	Malacostraca	Arthropoda	*Palaemonetes hobbsi*	0	0	0	0	0	0	0	0	0
Palaemonidae	Decapoda	Malacostraca	Arthropoda	*Palaemonetes kadiakensis*	0	0	0	0	0	0	0	0	0
Palaemonidae	Decapoda	Malacostraca	Arthropoda	*Palaemonetes lindsayi*	0	0	0	0	0	0	0	0	0
Palaemonidae	Decapoda	Malacostraca	Arthropoda	*Palaemonetes mexicanus*	0	0	0	0	0	0	0	0	0
Palaemonidae	Decapoda	Malacostraca	Arthropoda	*Palaemonetes suttkusi*	0	0	0	0	0	0	0	0	0
Palaemonidae	Decapoda	Malacostraca	Arthropoda	*Troglomexicanus huastecae*	0	0	0	0	0	0	0	0	0
Palaemonidae	Decapoda	Malacostraca	Arthropoda	*Troglomexicanus perezfarfantae*	0	0	0	0	0	0	0	0	0
Palaemonidae	Decapoda	Malacostraca	Arthropoda	*Troglomexicanus tamaulipasensis*	0	0	0	0	0	0	0	0	0
Pseudothelphusidae	Decapoda	Malacostraca	Arthropoda	*Lobithelphusa mexicana*	0	0	0	0	0	0	0	0	0
Pseudothelphusidae	Decapoda	Malacostraca	Arthropoda	*Spirothelphusa verticalis*	0	0	0	0	0	0	0	0	1
Pseudothelphusidae	Decapoda	Malacostraca	Arthropoda	*Odontothelphusa apicpac*	0	0	0	0	0	0	0	0	0
Pseudothelphusidae	Decapoda	Malacostraca	Arthropoda	*Odontothelphusa lacandona*	0	0	0	0	0	0	0	0	0
Pseudothelphusidae	Decapoda	Malacostraca	Arthropoda	*Odontothelphusa lacanjaensis*	0	0	0	0	0	0	0	0	0
Pseudothelphusidae	Decapoda	Malacostraca	Arthropoda	*Odontothelphusa maxillipes*	0	0	0	0	0	0	0	0	1
Pseudothelphusidae	Decapoda	Malacostraca	Arthropoda	*Odontothelphusa monodontis*	0	0	0	0	0	0	0	0	0
Pseudothelphusidae	Decapoda	Malacostraca	Arthropoda	*Odontothelphusa palenquensis*	0	0	0	0	0	0	0	0	0
Pseudothelphusidae	Decapoda	Malacostraca	Arthropoda	*Odontothelphusa toninae*	0	0	0	0	0	0	0	0	0
Pseudothelphusidae	Decapoda	Malacostraca	Arthropoda	*Phrygiopilus montebelloensis*	0	0	0	0	0	0	0	0	1
Pseudothelphusidae	Decapoda	Malacostraca	Arthropoda	*Phrygiopilus yoshibensis*	0	0	0	0	0	0	0	0	1
Pseudothelphusidae	Decapoda	Malacostraca	Arthropoda	*Potamocarcinus chajulensis*	0	0	0	0	0	0	0	0	0
Pseudothelphusidae	Decapoda	Malacostraca	Arthropoda	*Potamocarcinus hartmanni*	0	0	0	0	0	0	0	0	0
Pseudothelphusidae	Decapoda	Malacostraca	Arthropoda	*Potamocarcinus magnus*	0	0	0	0	0	0	0	0	1
Pseudothelphusidae	Decapoda	Malacostraca	Arthropoda	*Raddaus bocourti*	0	0	0	0	0	0	0	0	0
Pseudothelphusidae	Decapoda	Malacostraca	Arthropoda	*Raddaus tuberculatus*	0	0	0	0	0	0	0	0	0
Pseudothelphusidae	Decapoda	Malacostraca	Arthropoda	*Raddaus parazilchi mexicanus*	0	0	0	0	0	0	0	0	0
Pseudothelphusidae	Decapoda	Malacostraca	Arthropoda	*Typhlopseudothelphusa mocinoi*	0	0	0	0	0	0	0	0	0
Pseudothelphusidae	Decapoda	Malacostraca	Arthropoda	*Typhlopseudothelphusa hyba*	0	0	0	0	0	0	0	0	0
Pseudothelphusidae	Decapoda	Malacostraca	Arthropoda	*Villalobosus leptomelus*	0	0	0	0	0	0	0	0	0
Pseudothelphusidae	Decapoda	Malacostraca	Arthropoda	*Villalobosus lopezformenti*	0	0	0	0	0	0	0	0	1

Pseudothelphusidae	Decapoda	Malacostraca	Arthropoda	*Zilchia aspoeckorum*	0	0	0	0	0	0	0	0	0
Pseudothelphusidae	Decapoda	Malacostraca	Arthropoda	*Zilchia poglayeneuwalli*	0	0	0	0	0	0	0	0	0
Pseudothelphusidae	Decapoda	Malacostraca	Arthropoda	*Pseudothelphusa americana*	0	0	0	0	0	0	0	0	0
Pseudothelphusidae	Decapoda	Malacostraca	Arthropoda	*Pseudothelphusa belliana*	0	0	0	0	0	0	1	0	0
Pseudothelphusidae	Decapoda	Malacostraca	Arthropoda	*Pseudothelphusa digueti*	0	0	0	0	0	0	0	0	0
Pseudothelphusidae	Decapoda	Malacostraca	Arthropoda	*Pseudothelphusa dilatata*	0	0	0	0	1	0	0	0	0
Pseudothelphusidae	Decapoda	Malacostraca	Arthropoda	*Pseudothelphusa doenitzi*	0	0	0	0	0	0	0	0	1
Pseudothelphusidae	Decapoda	Malacostraca	Arthropoda	*Pseudothelphusa dugesi*	0	0	0	0	0	1	0	0	0
Pseudothelphusidae	Decapoda	Malacostraca	Arthropoda	*Pseudothelphusa galloi*	0	0	0	0	0	0	1	0	0
Pseudothelphusidae	Decapoda	Malacostraca	Arthropoda	*Pseudothelphusa granatensis*	0	0	0	0	0	1	0	0	0
Pseudothelphusidae	Decapoda	Malacostraca	Arthropoda	*Pseudothelphusa guerreroensis*	0	0	0	0	0	0	1	0	0
Pseudothelphusidae	Decapoda	Malacostraca	Arthropoda	*Pseudothelphusa hoffmanna*	0	0	0	0	0	0	1	0	0
Pseudothelphusidae	Decapoda	Malacostraca	Arthropoda	*Pseudothelphusa jouyi*	0	0	0	0	0	0	0	0	0
Pseudothelphusidae	Decapoda	Malacostraca	Arthropoda	*Pseudothelphusa leiophrys*	0	0	0	0	1	0	0	0	0
Pseudothelphusidae	Decapoda	Malacostraca	Arthropoda	*Pseudothelphusa lophophalus*	0	0	0	0	0	0	0	0	0
Pseudothelphusidae	Decapoda	Malacostraca	Arthropoda	*Pseudothelphusa mexicana*	0	0	0	0	0	1	0	0	0
Pseudothelphusidae	Decapoda	Malacostraca	Arthropoda	*Pseudothelphusa morelosis*	0	0	0	0	0	1	0	0	0
Pseudothelphusidae	Decapoda	Malacostraca	Arthropoda	*Pseudothelphusa nelsoni*	0	0	0	0	0	1	0	0	0
Pseudothelphusidae	Decapoda	Malacostraca	Arthropoda	*Pseudothelphusa parabelliana*	0	0	0	0	0	0	0	0	0
Pseudothelphusidae	Decapoda	Malacostraca	Arthropoda	*Pseudothelphusa pecki*	0	0	0	0	0	0	0	0	0
Pseudothelphusidae	Decapoda	Malacostraca	Arthropoda	*Pseudothelphusa seiferti*	0	0	0	1	0	0	0	0	0
Pseudothelphusidae	Decapoda	Malacostraca	Arthropoda	*Pseudothelphusa sulcifrons*	0	0	0	0	0	0	0	1	0
Pseudothelphusidae	Decapoda	Malacostraca	Arthropoda	*Pseudothelphusa terrestris*	0	0	0	1	0	0	0	0	0
Pseudothelphusidae	Decapoda	Malacostraca	Arthropoda	*Pseudothelphusa zongolicae*	0	0	0	0	0	0	0	0	0
Pseudothelphusidae	Decapoda	Malacostraca	Arthropoda	*Smalleyus tricristatus*	0	0	0	0	0	0	0	0	0
Pseudothelphusidae	Decapoda	Malacostraca	Arthropoda	*Tehuana diabolis*	0	0	0	0	0	0	0	0	0
Pseudothelphusidae	Decapoda	Malacostraca	Arthropoda	*Tehuana jacatepecensis*	0	0	0	0	0	0	0	0	1
Pseudothelphusidae	Decapoda	Malacostraca	Arthropoda	*Tehuana lamellifrons*	0	0	0	0	0	0	0	0	1
Pseudothelphusidae	Decapoda	Malacostraca	Arthropoda	*Tehuana lamothei*	0	0	0	0	0	0	0	0	0
Pseudothelphusidae	Decapoda	Malacostraca	Arthropoda	*Tehuana poglayenorum*	0	0	0	0	0	0	0	0	0
Pseudothelphusidae	Decapoda	Malacostraca	Arthropoda	*Tehuana veracruzana*	0	0	0	0	0	0	0	0	0
Pseudothelphusidae	Decapoda	Malacostraca	Arthropoda	*Epithelphusa chiapensis*	0	0	0	0	0	0	0	0	1
Pseudothelphusidae	Decapoda	Malacostraca	Arthropoda	*Epithelphusa mixtepensis*	0	0	0	0	0	0	0	0	0
Pseudothelphusidae	Decapoda	Malacostraca	Arthropoda	*Sylvathelphusa kalebi*	0	0	0	0	0	0	0	0	0
Pseudothelphusidae	Decapoda	Malacostraca	Arthropoda	*Sylvathelphusa cavernicola*	0	0	0	0	0	0	0	0	0
Pseudothelphusidae	Decapoda	Malacostraca	Arthropoda	*Tzotzithelphusa villarosalensis*	0	0	0	0	0	0	0	0	0
Poecilidae	Cyprinodontiformes	Actinopterygii	Chordata	*Belonesox belizanus*	0	0	0	0	0	0	0	0	0
Poecilidae	Cyprinodontiformes	Actinopterygii	Chordata	*Brachyrhaphis hartwegi*	0	0	0	0	0	0	0	0	1
Poecilidae	Cyprinodontiformes	Actinopterygii	Chordata	*Carlhubbsia kidderi*	0	0	0	0	0	0	0	0	0
Poecilidae	Cyprinodontiformes	Actinopterygii	Chordata	*Gambusia affinis*	1	0	0	0	0	0	0	0	0
Poecilidae	Cyprinodontiformes	Actinopterygii	Chordata	*Gambusia atrora*	0	0	0	0	0	0	0	0	0
Poecilidae	Cyprinodontiformes	Actinopterygii	Chordata	*Gambusia aurata*	0	0	0	0	0	0	0	0	0
Poecilidae	Cyprinodontiformes	Actinopterygii	Chordata	*Gambusia clarkhubbsi*	0	0	0	0	0	0	0	0	0
Poecilidae	Cyprinodontiformes	Actinopterygii	Chordata	*Gambusia eurystoma*	0	0	0	0	0	0	0	0	0
Poecilidae	Cyprinodontiformes	Actinopterygii	Chordata	*Gambusia hurtadoi*	0	0	0	0	0	0	0	0	0
Poecilidae	Cyprinodontiformes	Actinopterygii	Chordata	*Gambusia krumholzi*	0	0	0	0	0	0	0	0	0
Poecilidae	Cyprinodontiformes	Actinopterygii	Chordata	*Gambusia longispinis*	0	0	0	0	0	0	0	0	0
Poecilidae	Cyprinodontiformes	Actinopterygii	Chordata	*Gambusia marshi*	0	0	0	0	0	0	0	0	0
Poecilidae	Cyprinodontiformes	Actinopterygii	Chordata	*Gambusia panuco*	0	0	0	0	0	0	0	0	0
Poecilidae	Cyprinodontiformes	Actinopterygii	Chordata	*Gambusia regani*	0	0	0	0	0	0	0	0	0
Poecilidae	Cyprinodontiformes	Actinopterygii	Chordata	*Gambusia senilis*	0	0	0	0	0	0	0	0	0
Poecilidae	Cyprinodontiformes	Actinopterygii	Chordata	*Gambusia sexradiata*	0	0	0	0	0	0	0	0	0
Poecilidae	Cyprinodontiformes	Actinopterygii	Chordata	*Gambusia speciosa*	0	0	0	0	0	0	0	0	0
Poecilidae	Cyprinodontiformes	Actinopterygii	Chordata	*Gambusia vittata*	0	0	0	0	0	0	0	0	0
Poecilidae	Cyprinodontiformes	Actinopterygii	Chordata	*Gambusia yucatana*	0	0	0	0	0	0	0	0	0
Poecilidae	Cyprinodontiformes	Actinopterygii	Chordata	*Heterophallus echeagarayi*	0	0	0	0	0	0	0	0	0

Poecilidae	Cyprinodontiformes	Actinopterygii	Chordata	*Heterophallus milleri*	0	0	0	0	0	0	0	0	0
Poecilidae	Cyprinodontiformes	Actinopterygii	Chordata	*Heterophallus rachovii*	0	0	0	0	0	0	0	0	0
Poecilidae	Cyprinodontiformes	Actinopterygii	Chordata	*Phallichthys fairweatheri*	0	0	0	0	0	0	0	0	0
Poecilidae	Cyprinodontiformes	Actinopterygii	Chordata	*Poecilia butleri*	0	1	1	1	1	1	1	1	0
Poecilidae	Cyprinodontiformes	Actinopterygii	Chordata	*Poecilia catemaconis*	0	0	0	0	0	0	0	0	0
Poecilidae	Cyprinodontiformes	Actinopterygii	Chordata	*Poecilia chica*	0	0	1	0	0	0	0	0	0
Poecilidae	Cyprinodontiformes	Actinopterygii	Chordata	*Poecilia formosa*	0	0	0	0	0	0	0	0	0
Poecilidae	Cyprinodontiformes	Actinopterygii	Chordata	*Poecilia latipinna*	0	0	0	0	0	0	0	0	0
Poecilidae	Cyprinodontiformes	Actinopterygii	Chordata	*Poecilia latipunctata*	0	0	0	0	0	0	0	0	0
Poecilidae	Cyprinodontiformes	Actinopterygii	Chordata	*Poecilia mexicana*	0	0	0	0	0	0	0	0	0
Poecilidae	Cyprinodontiformes	Actinopterygii	Chordata	*Poecilia orri*	0	0	0	0	0	0	0	0	0
Poecilidae	Cyprinodontiformes	Actinopterygii	Chordata	*Poecilia petenensis*	0	0	0	0	0	0	0	0	0
Poecilidae	Cyprinodontiformes	Actinopterygii	Chordata	*Poecilia sphenops*	1	0	0	0	0	0	1	1	1
Poecilidae	Cyprinodontiformes	Actinopterygii	Chordata	*Poecilia sulphuraria*	0	0	0	0	0	0	0	0	0
Poecilidae	Cyprinodontiformes	Actinopterygii	Chordata	*Poecilia velifera*	0	0	0	0	0	0	0	0	0
Poecilidae	Cyprinodontiformes	Actinopterygii	Chordata	*Poeciliopsis baenschi*	0	0	0	0	1	0	0	0	0
Poecilidae	Cyprinodontiformes	Actinopterygii	Chordata	*Poeciliopsis balsas*	0	0	0	0	0	1	0	0	0
Poecilidae	Cyprinodontiformes	Actinopterygii	Chordata	*Poeciliopsis catemaco*	0	0	0	0	0	0	0	0	0
Poecilidae	Cyprinodontiformes	Actinopterygii	Chordata	*Poeciliopsis fasciata*	0	0	0	0	0	1	1	0	1
Poecilidae	Cyprinodontiformes	Actinopterygii	Chordata	*Poeciliopsis gracilis*	0	0	0	0	0	0	0	0	0
Poecilidae	Cyprinodontiformes	Actinopterygii	Chordata	*Poeciliopsis hnilickai*	0	0	0	0	0	0	0	0	0
Poecilidae	Cyprinodontiformes	Actinopterygii	Chordata	*Poeciliopsis infans*	0	0	1	1	1	1	1	0	0
Poecilidae	Cyprinodontiformes	Actinopterygii	Chordata	*Poeciliopsis latidens*	0	1	0	0	0	0	0	0	0
Poecilidae	Cyprinodontiformes	Actinopterygii	Chordata	*Poeciliopsis lucida*	0	1	1	0	0	0	0	0	0
Poecilidae	Cyprinodontiformes	Actinopterygii	Chordata	*Poeciliopsis lutzi*	0	0	0	0	0	0	0	0	0
Poecilidae	Cyprinodontiformes	Actinopterygii	Chordata	*Poeciliopsis monacha*	0	1	0	0	0	0	0	0	0
Poecilidae	Cyprinodontiformes	Actinopterygii	Chordata	*Poeciliopsis occidentalis*	0	1	0	0	0	0	0	0	0
Poecilidae	Cyprinodontiformes	Actinopterygii	Chordata	*Poeciliopsis pleurospilus*	0	0	0	0	0	0	0	1	1
Poecilidae	Cyprinodontiformes	Actinopterygii	Chordata	*Poeciliopsis presidionis*	0	1	0	0	0	0	0	0	0
Poecilidae	Cyprinodontiformes	Actinopterygii	Chordata	*Poeciliopsis prolifica*	0	1	1	0	0	0	0	0	0
Poecilidae	Cyprinodontiformes	Actinopterygii	Chordata	*Poeciliopsis scarlli*	0	0	0	0	0	1	0	0	0
Poecilidae	Cyprinodontiformes	Actinopterygii	Chordata	*Poeciliopsis turneri*	0	0	1	0	0	0	0	0	0
Poecilidae	Cyprinodontiformes	Actinopterygii	Chordata	*Poeciliopsis turrubarensis*	0	0	0	1	1	1	1	1	1
Poecilidae	Cyprinodontiformes	Actinopterygii	Chordata	*Poeciliopsis viriosa*	0	1	1	0	0	0	0	0	0
Poecilidae	Cyprinodontiformes	Actinopterygii	Chordata	*Priapella bonita*	0	0	0	0	0	0	0	0	0
Poecilidae	Cyprinodontiformes	Actinopterygii	Chordata	*Priapella chamulae*	0	0	0	0	0	0	0	0	0
Poecilidae	Cyprinodontiformes	Actinopterygii	Chordata	*Priapella compressa*	0	0	0	0	0	0	0	0	0
Poecilidae	Cyprinodontiformes	Actinopterygii	Chordata	*Priapella intermedia*	0	0	0	0	0	0	0	0	0
Poecilidae	Cyprinodontiformes	Actinopterygii	Chordata	*Priapella lacandonae*	0	0	0	0	0	0	0	0	0
Poecilidae	Cyprinodontiformes	Actinopterygii	Chordata	*Priapella olmecae*	0	0	0	0	0	0	0	0	0
Poecilidae	Cyprinodontiformes	Actinopterygii	Chordata	*Pseudoxiphophorus bimaculatus*	0	0	0	0	0	0	0	0	0
Poecilidae	Cyprinodontiformes	Actinopterygii	Chordata	*Pseudoxiphophorus jonesii*	0	0	0	0	0	0	0	0	0
Poecilidae	Cyprinodontiformes	Actinopterygii	Chordata	*Pseudoxiphophorus tuxtlaensis*	0	0	0	0	0	0	0	0	0
Poecilidae	Cyprinodontiformes	Actinopterygii	Chordata	*Xenodexia ctenolepis*	0	0	0	0	0	0	0	0	0
Poecilidae	Cyprinodontiformes	Actinopterygii	Chordata	*Xiphophorus alvarezi*	0	0	0	0	0	0	0	0	0
Poecilidae	Cyprinodontiformes	Actinopterygii	Chordata	*Xiphophorus clemenciae*	0	0	0	0	0	0	0	0	0
Poecilidae	Cyprinodontiformes	Actinopterygii	Chordata	*Xiphophorus couchianus*	0	0	0	0	0	0	0	0	0
Poecilidae	Cyprinodontiformes	Actinopterygii	Chordata	*Xiphophorus gordoni*	0	0	0	0	0	0	0	0	0
Poecilidae	Cyprinodontiformes	Actinopterygii	Chordata	*Xiphophorus hellerii*	1	0	0	0	0	0	0	1	0
Poecilidae	Cyprinodontiformes	Actinopterygii	Chordata	*Xiphophorus kallmani*	0	0	0	0	0	0	0	0	0
Poecilidae	Cyprinodontiformes	Actinopterygii	Chordata	*Xiphophorus maculatus*	1	0	0	0	0	0	0	0	0
Poecilidae	Cyprinodontiformes	Actinopterygii	Chordata	*Xiphophorus meyeri*	0	0	0	0	0	0	0	0	0
Poecilidae	Cyprinodontiformes	Actinopterygii	Chordata	*Xiphophorus variatus*	0	0	0	0	0	0	0	0	0
Poecilidae	Cyprinodontiformes	Actinopterygii	Chordata	*Xiphophorus xiphidium*	0	0	0	0	0	0	0	0	0
Derogenidae	Azygiida	Trematoda	Platyhelminthes	*Genarchella astyanactis*	0	0	0	0	0	1	0	0	0
Derogenidae	Azygiida	Trematoda	Platyhelminthes	*Genarchella isabellae*	0	0	0	0	0	0	0	0	0

Derogenidae	Azygiida	Trematoda	Platyhelminthes	*Genarchella tropica*	0	0	0	0	0	0	0	0	1
Haploporidae	Echinostomida	Trematoda	Platyhelminthes	*Alloglossidium corti*	0	1	0	0	0	0	0	0	0
Haploporidae	Echinostomida	Trematoda	Platyhelminthes	*Culuwiya cichlidorum*	0	0	0	0	0	0	0	0	1
Haploporidae	Echinostomida	Trematoda	Platyhelminthes	*Saccocoelioides chauhani*	0	0	0	0	0	0	0	0	0
Haploporidae	Echinostomida	Trematoda	Platyhelminthes	*Saccocoelioides lamothei*	0	0	0	0	0	0	1	0	0
Haploporidae	Echinostomida	Trematoda	Platyhelminthes	*Saccocoelioides sogandaresi*	0	0	0	0	1	1	1	0	1
Haploporidae	Echinostomida	Trematoda	Platyhelminthes	*Saccocoeliodes sp. (ex.: Eleotridae)*	0	0	0	0	0	0	1	0	0
Microscaphidiidae	Echinostomida	Trematoda	Platyhelminthes	*Octangioides ujati*	0	0	0	0	0	0	0	0	0
Allocreadiidae	Plagiorchiida	Trematoda	Platyhelminthes	*Auriculostoma astyanace*	0	0	0	0	0	0	0	0	0
Allocreadiidae	Plagiorchiida	Trematoda	Platyhelminthes	*Allocreadium lobatum*	0	0	0	0	0	0	0	0	0
Allocreadiidae	Plagiorchiida	Trematoda	Platyhelminthes	*Allocreadium mexicanum*	0	0	0	1	0	0	0	0	0
Allocreadiidae	Plagiorchiida	Trematoda	Platyhelminthes	*Crepidostomum cornutum*	0	0	0	0	0	0	0	0	0
Allocreadiidae	Plagiorchiida	Trematoda	Platyhelminthes	*Crepidostomum cooperi*	1	0	0	0	0	0	0	0	0
Allocreadiidae	Plagiorchiida	Trematoda	Platyhelminthes	*Creptotrema agonostomi*	0	0	1	0	1	1	0	0	0
Allocreadiidae	Plagiorchiida	Trematoda	Platyhelminthes	*Creptotrema sp.*	0	0	0	0	0	0	0	0	0
Allocreadiidae	Plagiorchiida	Trematoda	Platyhelminthes	*Margotrema bravoae*	0	0	0	0	1	0	0	0	0
Allocreadiidae	Plagiorchiida	Trematoda	Platyhelminthes	*Margotrema guillerminae*	0	0	0	0	0	1	0	0	0
Allocreadiidae	Plagiorchiida	Trematoda	Platyhelminthes	*Megalogonia ictaluri*	0	0	0	0	0	0	0	0	0
Allocreadiidae	Plagiorchiida	Trematoda	Platyhelminthes	*Paracreptotrema blancoi*	0	0	0	0	0	0	1	1	1
Allocreadiidae	Plagiorchiida	Trematoda	Platyhelminthes	*Paracreptotrema profundulusi*	0	0	0	0	0	0	0	1	1
Allocreadiidae	Plagiorchiida	Trematoda	Platyhelminthes	*Paracreptotrematina aguirrepequeñoi*	0	0	0	0	0	0	0	0	0
Allocreadiidae	Plagiorchiida	Trematoda	Platyhelminthes	*Polylekithum sp.*	0	0	0	0	0	0	1	0	0
Apocreadiidae	Plagiorchiida	Trematoda	Platyhelminthes	*Crassicutis cichlasomae*	0	0	0	0	0	0	1	0	1
Apocreadiidae	Plagiorchiida	Trematoda	Platyhelminthes	*Crassicutis choudhury*	0	0	0	1	0	0	0	0	0
Apocreadiidae	Plagiorchiida	Trematoda	Platyhelminthes	*Homalometron pallidum*	0	0	0	0	0	0	0	0	0
Cryptogonimidae	Plagiorchiida	Trematoda	Platyhelminthes	*Acanthostomum astorquii*	0	0	0	0	0	0	0	0	0
Cryptogonimidae	Plagiorchiida	Trematoda	Platyhelminthes	*Acanthostomum minima*	0	0	0	0	0	0	0	0	0
Cryptogonimidae	Plagiorchiida	Trematoda	Platyhelminthes	*Campechetrema herrerai*	0	0	0	0	0	0	0	0	0
Cryptogonimidae	Plagiorchiida	Trematoda	Platyhelminthes	*Campechetrema sp.*	0	0	0	0	0	0	0	0	0
Cryptogonimidae	Plagiorchiida	Trematoda	Platyhelminthes	*Neochasmus olmecus*	0	0	0	0	0	0	0	0	0
Cryptogonimidae	Plagiorchiida	Trematoda	Platyhelminthes	*Oligogonotylus manteri*	0	0	0	0	0	0	0	0	0
Cryptogonimidae	Plagiorchiida	Trematoda	Platyhelminthes	*Oligogonotylus mayae*	0	0	0	0	0	0	0	0	0
Cryptogonimidae	Plagiorchiida	Trematoda	Platyhelminthes	*Olmeca laurae*	0	0	0	0	0	0	0	0	0
Cryptogonimidae	Plagiorchiida	Trematoda	Platyhelminthes	*Paleocryptogonimus batallae*	0	0	0	0	0	0	0	0	0
Cryptogonimidae	Plagiorchiida	Trematoda	Platyhelminthes	*Tabascotrema verai*	0	0	0	0	0	0	0	0	0
Gorgoderidae	Plagiorchiida	Trematoda	Platyhelminthes	*Dendrorchis sp.*	0	0	0	0	1	0	1	0	0
Gorgoderidae	Plagiorchiida	Trematoda	Platyhelminthes	*Phyllodistomum centropomi*	0	0	0	0	0	0	0	0	0
Gorgoderidae	Plagiorchiida	Trematoda	Platyhelminthes	*Phyllodistomum lacustri*	0	0	0	0	0	0	0	0	0
Gorgoderidae	Plagiorchiida	Trematoda	Platyhelminthes	*Phyllodistomum sp. (ex.: Ciclidae)*	0	0	0	0	0	0	0	0	0
Gorgoderidae	Plagiorchiida	Trematoda	Platyhelminthes	*Phyllodistomum sp. (ex.: Profundulus)*	0	0	0	0	0	0	0	0	1
Macroderoididae	Plagiorchiida	Trematoda	Platyhelminthes	*Magnivitellinum simplex*	0	0	0	0	1	1	0	0	0
Macroderoididae	Plagiorchiida	Trematoda	Platyhelminthes	*Perezitrema bychowskyi*	0	0	0	0	0	0	0	0	0
Macroderoididae	Plagiorchiida	Trematoda	Platyhelminthes	*Wallinia chavarriae*	0	0	0	0	0	0	0	0	1
Callodistomidae	Strigeidida	Trematoda	Platyhelminthes	*Prosthenhystera caballeroi*	0	0	0	0	0	0	0	0	0
Callodistomidae	Strigeidida	Trematoda	Platyhelminthes	*Prosthenhystera obesa*	0	0	0	0	0	0	0	0	0
Microcotylidae	Mazocraeidea	Monogenea	Platyhelminthes	*Microcotyle spinicirrus*	0	0	0	0	0	0	0	0	0
Octomacridae	Mazocraeidea	Monogenea	Platyhelminthes	*Octomacrum mexicanum*	0	0	0	0	0	0	0	0	0
Dactylogyridae	Monopisthocotylea	Monogenea	Platyhelminthes	*Ameloblastella chavarriai*	0	0	0	0	0	0	0	0	1
Dactylogyridae	Monopisthocotylea	Monogenea	Platyhelminthes	*Aphanoblastella travassosi*	0	0	0	0	0	0	0	0	1
Dactylogyridae	Monopisthocotylea	Monogenea	Platyhelminthes	*Cichlidogyrus longicornis*	0	0	0	0	0	0	0	0	0
Dactylogyridae	Monopisthocotylea	Monogenea	Platyhelminthes	*Cichlidogyrus sclerosus*	0	0	0	0	0	0	0	0	0
Dactylogyridae	Monopisthocotylea	Monogenea	Platyhelminthes	*Cichlidogyrus tilapiae*	0	0	0	0	0	0	0	0	0
Dactylogyridae	Monopisthocotylea	Monogenea	Platyhelminthes	*Cleidodiscus sp. (ex.: Ictaluridae)*	0	0	0	0	0	1	0	0	0
Dactylogyridae	Monopisthocotylea	Monogenea	Platyhelminthes	*Characithecium costaricensis*	0	0	0	0	0	1	0	0	0
Dactylogyridae	Monopisthocotylea	Monogenea	Platyhelminthes	*Dactylogyrus sp. 1 (ex.: Gila)*	0	0	0	0	0	0	0	0	0
Dactylogyridae	Monopisthocotylea	Monogenea	Platyhelminthes	*Dactylogyrus sp. 2 (ex.: Gila)*	0	0	0	0	0	0	0	0	0

Dactylogyridae	Monopisthocotylea	Monogenea	Platyhelminthes	*Diaphorocleidus kabatai*	0	0	0	0	0	0	0	1	0
Dactylogyridae	Monopisthocotylea	Monogenea	Platyhelminthes	*Enterogyrus malmbergi*	0	0	0	0	0	0	0	0	0
Dactylogyridae	Monopisthocotylea	Monogenea	Platyhelminthes	*Guavinella tropica*	0	0	1	0	0	0	0	0	0
Dactylogyridae	Monopisthocotylea	Monogenea	Platyhelminthes	*Ligictaluridus mirabilis*	0	0	0	0	0	0	0	0	0
Dactylogyridae	Monopisthocotylea	Monogenea	Platyhelminthes	*Ligictaluridus pricei*	0	0	0	0	0	0	0	0	0
Dactylogyridae	Monopisthocotylea	Monogenea	Platyhelminthes	*Palombitrema heteroancistrium*	0	0	0	0	0	0	0	0	0
Dactylogyridae	Monopisthocotylea	Monogenea	Platyhelminthes	*Pavanelliella scaphiocotylus*	0	0	0	0	0	0	0	0	0
Dactylogyridae	Monopisthocotylea	Monogenea	Platyhelminthes	*Salsuginus neotropicalis*	0	0	0	0	0	0	0	0	0
Dactylogyridae	Monopisthocotylea	Monogenea	Platyhelminthes	*Salsuginus seculus*	0	0	0	0	0	0	0	0	0
Dactylogyridae	Monopisthocotylea	Monogenea	Platyhelminthes	*Salsuginus sp. (ex.: Goodeidae)*	0	0	0	0	0	1	0	0	0
Dactylogyridae	Monopisthocotylea	Monogenea	Platyhelminthes	*Salsuginus sp. (ex.: Poeciliidae)*	1	0	0	0	0	0	0	1	0
Dactylogyridae	Monopisthocotylea	Monogenea	Platyhelminthes	*Sciadicleithrum bravohollisae*	0	0	0	0	0	0	0	0	1
Dactylogyridae	Monopisthocotylea	Monogenea	Platyhelminthes	*Sciadicleithrum meekii*	0	0	0	0	0	0	0	0	0
Dactylogyridae	Monopisthocotylea	Monogenea	Platyhelminthes	*Sciadicleithrum mexicanum*	0	0	0	0	0	0	0	0	1
Dactylogyridae	Monopisthocotylea	Monogenea	Platyhelminthes	*Sciadicleithrum splendidae*	0	0	0	0	0	0	0	0	0
Dactylogyridae	Monopisthocotylea	Monogenea	Platyhelminthes	*Sciadicleithrum sp.*	0	0	0	1	0	0	0	0	0
Dactylogyridae	Monopisthocotylea	Monogenea	Platyhelminthes	*Urocleidoides reticulatus*	0	0	0	0	0	0	0	0	0
Dactylogyridae	Monopisthocotylea	Monogenea	Platyhelminthes	*Urocleidoides strombicirrus*	0	0	0	0	0	1	0	0	1
Diplectanidae	Monopisthocotylea	Monogenea	Platyhelminthes	*Neodiplectanum sp.*	0	0	0	0	0	0	0	0	0
Gyrodactylidae	Monopisthocotylea	Monogenea	Platyhelminthes	*Anacanthocotyle anacanthocotyle*	0	0	0	0	0	0	0	0	0
Gyrodactylidae	Monopisthocotylea	Monogenea	Platyhelminthes	*Gyrodactylus lamothei*	0	0	0	0	0	0	0	0	0
Gyrodactylidae	Monopisthocotylea	Monogenea	Platyhelminthes	*Gyrodactylus mexicanus*	0	0	0	0	0	0	0	0	0
Gyrodactylidae	Monopisthocotylea	Monogenea	Platyhelminthes	*Gyrodactylus neotropicalis*	0	0	0	0	0	0	0	0	0
Gyrodactylidae	Monopisthocotylea	Monogenea	Platyhelminthes	*Gyrodactylus spathulatus*	0	0	0	0	0	0	0	0	0
Gyrodactylidae	Monopisthocotylea	Monogenea	Platyhelminthes	*Gyrodactylus sp. 1 (ex.: Ciclidae)*	0	0	0	0	0	0	0	0	0
Gyrodactylidae	Monopisthocotylea	Monogenea	Platyhelminthes	*Gyrodactylus sp. 2 (ex.: Poeciliidae)*	1	0	0	1	0	1	0	1	1
Gyrodactylidae	Monopisthocotylea	Monogenea	Platyhelminthes	*Gyrodactylus sp. 3 (ex.: Eleotridae)*	0	0	0	0	0	0	0	0	0
Gyrodactylidae	Monopisthocotylea	Monogenea	Platyhelminthes	*Gyrodactylus sp. 4 (ex.: Rhamdia)*	0	0	0	0	0	0	0	0	0
Gyrodactylidae	Monopisthocotylea	Monogenea	Platyhelminthes	*Gyrodactylus sp. 5 (ex.: Gila)*	0	0	0	0	0	0	0	0	0
Gyrodactylidae	Monopisthocotylea	Monogenea	Platyhelminthes	*Gyrodactylus sp. 6 (ex.: Catostomus)*	0	0	0	0	0	0	0	0	0
Gyrodactylidae	Monopisthocotylea	Monogenea	Platyhelminthes	*Gyrodactylus sp. 7 (ex.: Notropis)*	0	0	0	0	0	0	0	0	0
Gyrodactylidae	Monopisthocotylea	Monogenea	Platyhelminthes	*Gyrodactylus sp. 8 (ex.: Campostoma)*	0	0	0	0	0	0	0	0	0
Gyrodactylidae	Monopisthocotylea	Monogenea	Platyhelminthes	*Gyrodactylus sp. 9 (ex.: Astyanax)*	0	0	0	0	0	0	0	0	0
Caryophyllaeidae	Caryophyllidea	Cestoda	Platyhelminthes	*Isoglaridacris sp.*	0	0	0	0	0	0	0	0	0
Monticelliidae	Proteocephalidea	Cestoda	Platyhelminthes	*Amphoteromorphus sp.*	0	0	0	0	0	0	0	0	0
Monticelliidae	Proteocephalidea	Cestoda	Platyhelminthes	*Monticellia ophisterni*	0	0	0	0	0	0	0	0	0
Proteocephalidae	Proteocephalidea	Cestoda	Platyhelminthes	*Corallobothrium fimbriatum*	0	1	0	0	0	1	0	0	0
Proteocephalidae	Proteocephalidea	Cestoda	Platyhelminthes	*Megathylacoides giganteum*	0	0	0	0	0	1	0	0	0
Proteocephalidae	Proteocephalidea	Cestoda	Platyhelminthes	*Megathylacoides lamothei*	0	0	0	0	0	1	0	0	0
Proteocephalidae	Proteocephalidea	Cestoda	Platyhelminthes	*Proteocephalus brooksi*	0	0	0	0	0	0	0	0	0
Proteocephalidae	Proteocephalidea	Cestoda	Platyhelminthes	*Proteocephalus chamelensis*	0	0	1	0	0	0	0	0	0
Proteocephalidae	Proteocephalidea	Cestoda	Platyhelminthes	*Proteocephalus longicollis*	0	0	0	0	0	0	0	0	0
Proteocephalidae	Proteocephalidea	Cestoda	Platyhelminthes	*Proteocephalus singularis*	0	0	0	0	0	0	0	0	0
Proteocephalidae	Proteocephalidea	Cestoda	Platyhelminthes	*Proteocephalus sp. (ex.: Ictaluridae)*	0	0	0	0	0	0	0	0	0
Bothriocephalidae	Pseudophyllidea	Cestoda	Platyhelminthes	*Bothriocephalus acheilognathi*	1	0	0	1	1	1	0	1	0
Bothriocephalidae	Pseudophyllidea	Cestoda	Platyhelminthes	*Bothriocephalus cuspidatus*	0	0	0	0	0	0	0	0	0
Bothriocephalidae	Pseudophyllidea	Cestoda	Platyhelminthes	*Bothriocephalus pearsei*	0	0	0	0	0	0	0	0	0
Atactorhynchidae	Neoechinorhynchida	Eoacanthocephala	Acanthocephala	*Atactorhynchus duranguensis*	0	0	0	0	0	0	0	0	0
Neoechinorhynchidae	Neoechinorhynchida	Eoacanthocephala	Acanthocephala	*Gracilisentis gracilisentis*	0	0	0	0	0	0	0	0	0
Neoechinorhynchidae	Neoechinorhynchida	Eoacanthocephala	Acanthocephala	*Neoechinorhynchus brentnickoli*	1	0	1	0	0	0	1	0	0
Neoechinorhynchidae	Neoechinorhynchida	Eoacanthocephala	Acanthocephala	*Neoechinorhynchus chimalapensis*	0	0	0	0	0	0	0	0	0
Neoechinorhynchidae	Neoechinorhynchida	Eoacanthocephala	Acanthocephala	*Neoechinorhynchus golvani*	0	0	0	1	0	1	0	0	0
Neoechinorhynchidae	Neoechinorhynchida	Eoacanthocephala	Acanthocephala	*Neoechinorhynchus sp. (ex.: Eleotridae)*	0	0	0	0	0	0	0	0	0
Neoechinorhynchidae	Neoechinorhynchida	Eoacanthocephala	Acanthocephala	*Neoechinorhynchus sp,*	0	0	0	0	0	0	0	0	0
Neoechinorhynchidae	Neoechinorhynchida	Eoacanthocephala	Acanthocephala	*Octospiniferoides chandleri*	0	0	0	0	0	0	0	0	0
Neoechinorhynchidae	Neoechinorhynchida	Eoacanthocephala	Acanthocephala	*Pomphorhynchus bulbocolli*	0	0	0	0	0	0	0	0	0

Echinorhynchidae	Echinorhynchida	Palaeacanthocephala	Acanthocephala	*Acanthocephalus amini*	0	0	0	0	0	0	0	0	0
Capillariidae	Enoplida	Adenophorea	Nematoda	*Capillaria cyprinodonticola*	0	0	0	0	1	1	0	0	0
Capillariidae	Enoplida	Adenophorea	Nematoda	*Capillaria (H.) cichlasomae*	0	0	0	0	0	0	0	0	0
Capillariidae	Enoplida	Adenophorea	Nematoda	*Capillostrongyloides sp.*	0	0	0	0	0	0	0	0	0
Capillariidae	Enoplida	Adenophorea	Nematoda	*Paracapillaria rhamdiae*	0	0	0	0	0	0	0	0	1
Capillariidae	Enoplida	Adenophorea	Nematoda	*Paracapillaria teixeirafreitasi*	0	0	1	0	0	0	0	0	0
Capillariidae	Enoplida	Adenophorea	Nematoda	*Paracapillaroides agonostomi*	0	0	1	0	0	0	0	0	0
Capillariidae	Enoplida	Adenophorea	Nematoda	*Pseudocapillaria (I.) ophisterni*	0	0	0	0	0	0	0	0	0
Capillariidae	Enoplida	Adenophorea	Nematoda	*Pseudocapillaria tomentosa*	0	0	0	0	0	0	0	0	0
Capillariidae	Enoplida	Adenophorea	Nematoda	*Pseudocapillaria yucatanensis*	0	0	0	0	0	0	0	0	0
Cystoopsidae	Enoplida	Adenophorea	Nematoda	*Cystoopsis atractostei*	0	0	0	0	0	0	0	0	0
Anisakidae	Ascaridida	Secernentea	Nematoda	*Goezia nonipapillata*	0	0	0	0	0	1	0	0	0
Anisakidae	Ascaridida	Secernentea	Nematoda	*Goezia sp. (ex.: Ictaluridae)*	0	0	0	0	0	1	0	0	0
Anisakidae	Ascaridida	Secernentea	Nematoda	*Hysterothylacium cenotae*	0	0	0	0	0	0	0	0	1
Atractidae	Ascaridina	Secernentea	Nematoda	*Atractis bravoae*	0	0	0	0	0	1	0	0	0
Atractidae	Ascaridina	Secernentea	Nematoda	*Atractis vidali*	0	0	0	0	0	0	0	0	0
Atractidae	Ascaridina	Secernentea	Nematoda	*Orientatractis campechensis*	0	0	0	0	0	0	0	0	0
Atractidae	Ascaridina	Secernentea	Nematoda	*Orientatractis chiapasensis*	0	0	0	0	0	0	0	0	0
Camallanidae	Camallanida	Secernentea	Nematoda	*Procamallanus (S.) gobiomori*	0	0	1	0	0	0	0	0	0
Camallanidae	Camallanida	Secernentea	Nematoda	*Procamallanus (S.) jaliscensis*	0	0	1	0	1	0	0	0	0
Camallanidae	Camallanida	Secernentea	Nematoda	*Procamallanus (S.) neocaballeroi*	0	0	0	0	0	0	0	0	0
Camallanidae	Camallanida	Secernentea	Nematoda	*Procamallanus (S.) rebecae*	0	0	0	0	0	0	0	0	1
Cosmocercidae	Ascaridida	Secernentea	Nematoda	*Raillietnema kritscheri*	0	0	0	0	0	1	0	0	0
Cucullanidae	Ascaridida	Secernentea	Nematoda	*Cucullanus angeli*	0	0	0	1	0	1	0	0	0
Cucullanidae	Ascaridida	Secernentea	Nematoda	*Cucullanus (Cucullanus) caballeroi*	0	0	0	0	0	0	0	0	0
Cucullanidae	Ascaridida	Secernentea	Nematoda	*Cucullanus mexicanus*	0	0	0	0	0	0	0	0	0
Cucullanidae	Ascaridida	Secernentea	Nematoda	*Dichelyne mexicanus*	0	0	0	0	0	0	0	0	0
Cucullanidae	Ascaridida	Secernentea	Nematoda	*Neocucullanus neocucullanus*	0	0	0	0	0	0	0	0	0
Cystidicolidae	Spirurida	Secernentea	Nematoda	*Spinitectus agonostomi*	0	0	0	0	1	0	0	0	0
Cystidicolidae	Spirurida	Secernentea	Nematoda	*Spinitectus humbertoi*	0	0	0	0	0	0	1	0	1
Cystidicolidae	Spirurida	Secernentea	Nematoda	*Spinitectus mariaisabelae*	0	0	0	0	0	0	0	0	0
Cystidicolidae	Spirurida	Secernentea	Nematoda	*Spinitectus mexicanus*	0	0	0	0	0	0	0	0	0
Cystidicolidae	Spirurida	Secernentea	Nematoda	*Spinitectus osorioi*	0	0	0	0	0	0	0	0	0
Cystidicolidae	Spirurida	Secernentea	Nematoda	*Spinitectus tabascoensis*	0	0	0	0	0	0	0	0	0
Daniconematidae	Spirurida	Secernentea	Nematoda	*Mexiconema cichlasomae*	0	0	0	0	0	0	0	0	0
Philometridae	Camallanida	Secernentea	Nematoda	*Neophilometroides caudatus*	0	0	0	0	0	0	0	0	0
Philometridae	Camallanida	Secernentea	Nematoda	*Philometra ophisterni*	0	0	0	0	0	0	0	0	0
Philometridae	Camallanida	Secernentea	Nematoda	*Philometra poblana*	0	0	0	0	0	1	0	0	0
Philometridae	Camallanida	Secernentea	Nematoda	*Philometridae gen. sp.*	0	0	0	0	0	0	0	0	0
Quimperiidae	Ascaridida	Secernentea	Nematoda	*Gibsonnema ophisterni*	0	0	0	0	0	0	0	0	0
Rhabdochonidae	Spirurida	Secernentea	Nematoda	*Beaninema nayaritense*	0	0	0	1	0	0	0	0	0
Rhabdochonidae	Spirurida	Secernentea	Nematoda	*Rhabdochona acuminata*	0	0	0	0	0	0	0	0	0
Rhabdochonidae	Spirurida	Secernentea	Nematoda	*Rhabdochona ahuehuellensis*	0	0	0	0	1	1	0	0	0
Rhabdochonidae	Spirurida	Secernentea	Nematoda	*Rhabdochona canadensis*	0	0	0	0	0	1	0	1	0
Rhabdochonidae	Spirurida	Secernentea	Nematoda	*Rhabdochona cascadilla*	1	0	0	0	0	0	0	0	0
Rhabdochonidae	Spirurida	Secernentea	Nematoda	*Rhabdochona catostomi*	0	0	0	0	0	0	0	0	0
Rhabdochonidae	Spirurida	Secernentea	Nematoda	*Rhabdochona chimalapensis*	0	0	0	0	0	0	0	0	0
Rhabdochonidae	Spirurida	Secernentea	Nematoda	*Rhabdochona guerreroensis*	0	0	0	0	1	0	1	0	0
Rhabdochonidae	Spirurida	Secernentea	Nematoda	*Rhabdochona kidderi*	0	0	0	1	1	1	0	0	1
Rhabdochonidae	Spirurida	Secernentea	Nematoda	*Rhabdochona lichtenfelsi*	0	0	0	0	1	1	0	0	0
Rhabdochonidae	Spirurida	Secernentea	Nematoda	*Rhabdochona mexicana*	0	0	0	0	1	1	0	1	0
Rhabdochonidae	Spirurida	Secernentea	Nematoda	*Rhabdochona salgadoi*	0	0	0	0	0	0	1	1	1
Rhabdochonidae	Spirurida	Secernentea	Nematoda	*Rhabdochona xiphophori*	0	0	0	1	1	1	0	0	1

Chiapas Pacífico	Río Bravo	Lerma	Cuatro Ciénegas	Manantiales Durango	Río San Fernando	Pánuco	Tuxpan	Chimalapas	Papaloapan	Tabasco	Grijalva-Usumacinta	Yucatán
0	0	0	0	0	0	0	0	0	0	0	0	1
0	0	0	0	0	0	0	0	0	0	0	1	0
0	0	0	0	0	0	0	0	0	0	0	1	0
0	0	0	0	0	0	0	0	0	0	0	1	0
0	0	0	0	0	0	0	0	0	0	0	1	0
0	1	0	0	0	1	0	1	0	1	1	1	1
0	0	0	0	0	0	0	0	0	0	0	1	0
1	0	0	0	0	0	0	0	0	0	0	0	0
0	1	0	0	0	1	1	1	0	1	1	1	1
0	0	0	0	0	0	0	0	0	1	0	0	0
0	0	0	0	0	0	0	0	0	0	0	0	0
0	0	0	1	0	1	1	1	0	1	1	1	1
1	0	0	0	0	0	0	0	0	1	1	0	0
0	0	0	0	0	0	0	0	0	1	0	0	0
0	0	0	0	0	0	0	0	0	1	0	0	0
0	0	0	0	0	0	0	0	0	0	0	0	0
0	0	0	0	0	0	0	0	0	1	0	0	0
1	0	0	0	0	0	0	0	0	0	0	0	0
0	1	0	0	0	0	0	0	0	0	0	0	0
1	1	1	0	0	1	1	1	0	1	1	0	1
0	0	0	0	0	0	0	0	0	0	0	1	0
1	0	1	0	0	0	0	0	0	0	0	0	0
0	0	0	0	0	0	0	0	0	1	0	0	0
0	0	0	0	0	0	0	0	0	1	0	0	0
0	0	0	0	0	0	0	0	0	0	0	1	0
0	0	0	0	0	0	0	0	0	1	0	0	0
0	0	0	0	0	1	0	0	0	0	0	0	0
0	1	0	1	0	0	0	0	0	0	0	0	0
0	0	0	0	0	0	0	1	0	0	0	0	0
0	0	0	0	0	0	0	1	0	0	0	0	0
0	1	0	1	0	0	0	0	0	0	0	0	0
0	0	0	0	0	0	0	1	0	0	0	0	0
0	0	0	0	0	0	0	1	0	0	0	0	0
0	0	0	0	0	0	0	1	0	0	0	0	0
0	0	0	0	0	0	0	0	0	1	0	0	0
0	0	0	0	0	0	0	0	0	0	0	0	0
0	0	0	0	0	0	0	0	0	0	0	1	0
0	0	0	0	0	0	0	0	0	0	0	1	0
0	0	0	0	0	0	0	0	0	0	0	1	0
0	0	0	0	0	0	0	0	0	1	0	0	0
0	0	0	0	0	0	0	0	0	0	0	1	0
0	0	0	0	0	0	0	0	0	0	0	1	0
0	0	0	0	0	0	0	0	0	0	0	1	0
0	0	0	0	0	0	0	0	0	0	0	0	0
0	0	0	0	0	0	0	0	0	0	0	0	0
0	0	0	0	0	0	0	0	0	0	0	1	0
0	0	0	0	0	0	0	0	0	0	0	1	0
1	0	0	0	0	0	0	0	0	0	0	0	0
0	0	0	0	0	0	0	0	0	0	0	1	0
0	0	0	0	0	0	0	0	0	0	0	1	0
0	0	0	0	0	0	0	0	0	0	0	1	0
0	0	0	0	0	0	0	0	0	0	0	1	0
0	0	0	0	0	0	0	0	0	0	0	1	0
0	0	0	0	0	0	0	0	0	1	0	0	0
0	0	0	0	0	0	0	0	0	0	0	0	0

0	0	0	0	0	0	0	0	0	0	0	1	0
0	0	0	0	0	0	0	0	0	0	0	1	0
0	0	0	0	0	0	1	0	0	0	0	0	0
0	0	0	0	0	0	0	0	0	0	0	0	0
0	0	1	0	0	0	0	0	0	0	0	0	0
0	0	0	0	0	0	0	0	0	0	0	0	0
0	0	0	0	0	0	0	0	0	0	0	0	0
0	0	0	0	0	0	0	0	0	0	0	0	0
0	0	0	0	0	0	0	0	0	0	0	0	0
0	0	0	0	0	0	0	0	0	0	0	0	0
0	0	0	0	0	0	0	0	0	0	0	0	0
0	0	0	0	0	0	0	0	0	0	0	0	0
0	0	1	0	0	0	0	0	0	0	0	0	0
0	0	0	0	0	0	0	0	0	0	0	0	0
0	0	0	0	1	0	0	0	0	0	0	0	0
0	0	0	0	0	0	0	0	0	0	0	0	0
0	0	0	0	0	0	0	0	0	0	0	0	0
0	0	0	0	0	0	0	0	0	0	0	0	0
0	0	0	0	0	0	0	0	0	1	0	0	0
0	0	0	0	0	0	0	0	0	1	0	0	0
0	0	0	0	0	0	0	0	0	0	0	0	0
0	0	0	0	0	0	0	0	0	0	0	0	0
0	0	0	0	0	0	0	0	0	0	0	0	0
0	0	0	0	0	0	0	0	0	1	0	0	0
0	0	0	0	0	0	0	0	0	1	0	0	0
0	0	0	0	0	0	0	0	0	1	0	0	0
0	0	0	0	0	0	0	0	0	0	0	0	0
0	0	0	0	0	0	0	0	0	0	0	0	0
0	0	0	0	0	0	0	0	1	0	0	0	0
0	0	0	0	0	0	0	0	0	1	0	0	0
0	0	0	0	0	0	0	0	0	1	0	0	0
0	0	0	0	0	0	0	0	0	0	0	0	0
1	0	0	0	0	0	0	0	0	0	0	0	0
0	0	0	0	0	0	0	0	0	0	0	1	0
0	0	0	0	0	0	0	0	0	0	0	1	0
0	0	0	0	0	0	0	0	0	0	0	1	0
0	0	0	0	0	0	0	0	1	1	1	1	1
1	0	0	0	0	0	0	0	0	0	0	0	0
0	0	0	0	0	0	0	0	0	0	0	1	1
0	1	0	0	0	0	1	1	0	0	0	0	0
0	0	0	0	0	0	1	0	0	0	0	0	0
0	0	0	0	0	1	1	0	0	0	0	0	0
0	1	0	0	0	0	0	0	0	0	0	0	0
0	0	0	0	0	0	0	0	0	0	0	1	0
0	1	0	0	0	0	0	0	0	0	0	0	0
0	1	0	0	0	0	0	0	0	0	0	0	0
0	0	0	1	0	0	0	0	0	0	0	0	0
0	0	0	1	0	0	0	0	0	0	0	0	0
0	0	0	0	0	0	1	1	0	0	0	0	0
0	0	0	0	0	1	1	1	0	0	0	0	0
0	1	0	0	1	0	0	0	0	0	0	0	0
0	0	0	0	0	0	0	0	0	1	1	1	0
0	1	0	0	0	0	0	0	0	0	0	0	0
0	0	0	0	0	0	1	0	0	0	0	0	0
0	0	0	0	0	0	0	0	1	0	1	1	1
0	0	0	0	0	0	0	0	0	0	1	1	0

0	0	0	0	0	0	0	0	0	0	0	1	0
0	0	0	0	0	0	0	0	0	1	0	0	0
1	0	0	0	0	0	0	0	0	0	1	1	1
0	0	0	0	0	0	0	0	0	0	0	0	0
0	0	0	0	0	0	0	0	0	1	0	0	0
0	0	0	0	0	0	0	0	0	0	0	0	0
0	1	0	0	0	1	1	1	0	0	0	0	0
0	1	0	0	0	1	1	1	0	0	0	0	0
0	0	0	0	0	0	1	0	0	0	0	0	0
0	1	0	0	0	1	1	1	1	1	1	0	1
0	0	0	0	0	0	0	0	0	0	0	0	1
0	0	0	0	0	0	0	0	0	0	0	1	1
1	0	0	0	0	0	0	0	0	0	0	0	0
0	0	0	0	0	0	0	0	0	0	0	1	0
0	0	0	0	0	0	0	0	0	0	0	0	1
0	0	0	0	0	0	0	0	0	0	0	0	0
0	0	0	0	0	0	0	0	0	0	0	0	0
0	0	0	0	0	0	0	0	0	1	0	0	0
1	0	0	0	0	0	0	0	0	0	0	0	0
1	0	0	0	0	0	0	0	0	1	0	0	0
0	0	0	0	0	0	0	0	0	0	0	1	0
0	0	1	0	0	0	0	0	0	0	0	0	0
0	0	0	0	0	0	0	0	0	0	0	0	0
0	0	0	0	0	0	0	0	0	0	0	0	0
0	0	0	0	0	0	0	0	0	0	0	1	0
0	0	0	0	0	0	0	0	0	0	0	0	0
0	0	0	0	0	0	0	0	0	0	0	0	0
0	0	0	0	0	0	0	0	0	0	0	0	0
0	0	0	0	0	0	0	0	0	0	0	0	0
0	0	0	0	0	0	0	0	0	0	0	0	0
0	0	0	0	0	0	0	0	0	0	0	0	0
0	0	0	0	0	0	0	0	0	0	0	0	0
1	0	0	0	0	0	0	0	0	0	0	0	0
0	0	0	0	0	0	0	0	0	0	0	0	0
0	0	0	0	0	0	0	0	0	1	0	0	0
0	0	0	0	0	0	0	0	0	0	0	1	0
0	0	0	0	0	0	0	0	0	0	0	1	0
0	0	0	0	0	0	0	0	0	0	1	0	0
0	0	0	0	0	0	0	0	0	0	0	1	0
0	0	0	0	0	0	0	0	0	1	0	0	0
0	0	0	0	0	0	0	0	0	1	1	1	1
0	0	0	0	0	0	1	1	0	0	0	0	0
0	0	0	0	0	0	0	0	0	1	0	0	0
0	0	0	0	0	0	0	0	0	0	0	1	0
0	0	0	0	0	0	0	0	0	0	0	1	0
0	0	0	0	0	0	0	0	0	1	0	0	0
0	1	0	0	0	0	0	0	0	0	0	0	0
0	0	0	1	0	0	0	0	0	0	0	0	0
0	0	0	0	0	0	0	0	0	1	1	1	0
0	0	0	0	0	0	0	0	0	1	0	0	0
0	0	0	0	0	0	0	0	1	1	1	1	0
0	0	0	0	0	0	0	0	0	1	0	0	0
0	0	0	0	0	0	1	0	0	0	0	0	0
0	0	0	0	0	1	0	0	0	0	0	0	0
0	0	0	0	0	1	1	0	1	0	0	1	1
0	0	0	1	0	0	0	0	1	1	1	1	1

0	0	0	0	0	0	0	0	0	1	1	1	1
0	0	1	0	1	0	1	0	0	0	0	0	0
0	0	0	1	0	0	0	0	1	0	1	1	1
0	0	0	0	0	0	0	0	0	1	0	0	0
0	0	0	0	0	0	0	0	0	0	0	0	0
1	0	0	0	0	0	1	0	0	1	1	1	1
0	0	0	0	0	0	0	0	0	1	0	0	0
0	0	0	0	0	0	0	0	0	0	1	1	1
0	0	0	0	0	0	0	0	1	0	0	1	0
0	0	1	0	0	0	0	0	0	0	0	0	0
0	0	1	0	1	0	0	0	0	0	0	0	0
0	0	1	0	0	0	0	0	0	0	0	0	0
0	0	0	0	1	0	0	0	0	0	0	0	0
0	0	0	0	0	0	0	0	1	1	0	0	0
1	0	0	0	0	0	0	0	0	0	0	1	0
0	0	1	0	1	0	0	0	0	0	0	0	0
0	0	1	0	1	0	0	0	0	0	0	0	0
0	1	0	0	0	0	0	0	0	0	0	0	0
0	0	0	0	0	0	0	0	1	0	0	0	0
0	0	0	0	0	0	0	0	0	0	0	0	0
0	1	0	1	0	0	1	0	0	0	0	1	0
0	0	0	0	0	0	0	0	0	0	0	0	0
0	1	0	1	0	0	1	0	1	1	1	1	1
0	0	0	0	0	0	0	0	0	0	0	0	0
0	0	0	0	0	0	0	0	0	0	1	1	1
0	0	0	0	0	0	0	0	0	0	1	0	1
0	0	0	0	0	0	0	0	0	1	1	1	1
0	0	0	0	0	0	0	0	0	0	1	1	0
0	0	0	0	0	0	0	1	0	0	0	0	0
0	0	0	0	0	0	0	0	0	0	1	0	0
0	0	0	0	0	0	0	0	0	1	1	1	1
0	0	0	0	0	0	0	0	0	0	0	0	1
0	0	0	0	0	0	0	0	0	0	1	1	0
0	0	0	0	0	0	0	0	0	0	1	0	0
0	0	0	0	0	0	0	0	0	0	1	1	1
0	0	0	0	0	0	0	0	0	1	0	0	0
0	0	0	0	0	0	0	0	0	1	0	0	0
0	1	1	0	1	0	0	1	0	0	0	0	0
0	0	0	0	0	0	0	0	0	0	1	1	0
0	0	0	0	0	0	0	0	0	0	0	0	0
1	1	0	0	1	0	1	0	0	1	0	1	1
0	0	0	0	0	0	0	0	0	0	1	0	0
0	0	0	1	0	0	0	0	1	1	0	0	0
0	1	0	0	0	0	0	0	0	0	0	0	0
0	0	0	0	0	0	0	0	0	1	1	0	1
0	1	0	0	0	0	0	0	0	0	0	0	0
0	0	1	0	0	0	0	0	0	0	0	0	0
1	0	0	0	0	0	0	0	0	1	1	0	1
1	0	0	0	0	0	0	0	1	1	1	0	1
0	0	0	0	0	0	0	0	0	1	0	0	0
0	0	0	0	1	0	0	0	0	1	0	0	1
0	0	0	0	0	0	0	0	0	1	0	0	0
0	0	0	0	0	0	0	0	0	0	0	0	0
1	0	0	0	1	0	0	0	0	1	1	1	1
0	0	0	0	1	0	0	0	0	0	0	0	0
0	0	0	0	1	0	0	0	0	0	0	0	0

0	0	0	0	0	0	0	0	0	0	0	0	1
0	0	0	0	0	0	0	0	0	0	1	0	0
0	0	0	0	0	0	0	0	1	1	1	0	0
0	0	0	0	1	0	0	0	0	0	1	0	0
0	0	0	0	1	0	0	0	0	0	0	0	0
1	0	0	0	0	0	0	0	0	0	1	0	1
0	0	0	0	0	0	0	0	0	0	0	0	1
0	0	0	0	0	0	0	0	0	0	0	0	1
0	0	0	0	0	0	0	0	0	0	1	0	0
0	0	1	0	1	0	0	0	0	0	0	0	0
0	0	0	0	0	0	0	0	1	0	0	0	0
0	0	0	0	0	0	1	0	0	1	1	1	1
0	0	0	0	0	0	0	0	0	0	1	1	1
1	0	0	0	0	0	0	0	0	0	1	1	1
0	0	0	0	0	0	0	0	0	0	1	1	1
0	0	0	0	0	0	1	0	1	0	0	1	0
0	0	0	0	0	0	0	0	0	0	1	0	0
1	0	0	0	1	0	1	0	1	0	1	1	0
0	0	0	0	0	0	0	0	0	0	1	0	0
0	0	0	1	0	0	0	0	0	1	0	0	1
0	0	1	0	0	0	0	0	0	0	0	0	0
0	0	1	0	0	0	0	0	0	0	0	0	0
0	0	0	0	0	0	0	0	0	0	0	0	1
0	0	0	0	1	0	0	0	0	0	0	0	0
0	0	0	0	0	0	0	0	1	1	1	1	1
0	0	0	0	0	0	0	0	1	1	0	1	1
1	0	0	0	0	0	0	0	0	1	0	0	0
0	0	0	0	0	0	0	0	0	1	0	1	0
0	0	0	0	1	0	0	0	0	0	0	0	0
0	0	0	0	1	0	0	0	0	0	0	0	0
0	0	0	0	1	0	0	0	0	0	0	0	0
0	0	0	0	1	0	0	0	0	0	0	0	0
0	0	0	0	1	0	0	0	0	0	0	0	0
0	0	0	0	1	0	0	0	0	0	0	0	0
0	0	0	0	0	0	0	0	0	0	1	0	0
0	0	0	0	0	0	0	0	0	1	0	0	0
0	1	1	0	1	1	0	1	0	0	0	0	0
0	1	1	0	1	0	0	1	0	0	0	0	0
0	0	0	0	0	0	0	0	0	1	1	0	0
0	0	0	0	0	0	0	0	0	1	0	0	1
0	0	0	0	0	0	0	0	0	0	0	0	0
0	0	1	0	0	0	0	0	0	0	0	0	0
0	0	0	0	0	0	0	0	0	0	1	0	0
0	0	0	0	0	0	0	0	0	0	1	1	0
0	0	1	0	1	0	1	0	1	0	1	1	1
0	0	0	0	0	0	0	0	0	1	0	0	0
0	0	0	0	0	0	0	0	0	0	0	0	1
0	0	0	0	1	0	0	0	0	0	0	0	0
0	0	0	0	0	0	0	0	0	0	0	1	0
0	0	0	0	0	0	0	0	0	0	0	0	0
0	0	0	0	0	0	0	0	1	0	0	0	0
1	0	0	0	0	0	1	0	0	1	1	1	1
0	0	0	0	0	0	0	0	0	1	1	0	0
0	0	0	0	0	0	1	0	0	0	0	0	0
0	0	0	0	0	0	0	0	0	1	1	1	1
0	0	0	0	1	0	0	0	0	0	0	0	0

0	0	0	0	0	0	0	0	0	0	0	0	1
0	0	0	0	0	0	1	0	0	0	0	0	0
0	0	0	0	0	0	0	0	0	0	0	0	1
0	0	0	0	0	0	0	0	0	0	0	0	1
0	0	0	0	0	0	0	0	0	0	0	0	1
0	0	0	0	0	0	1	0	0	1	1	0	1
0	0	0	0	0	0	0	0	0	0	0	0	0
0	0	0	0	0	0	0	0	1	1	0	0	0
0	0	1	0	0	0	1	0	0	0	0	0	0
0	0	0	0	0	0	0	0	0	0	0	0	1
0	0	0	0	0	0	0	0	0	0	1	0	0
0	0	0	0	0	0	0	0	0	0	0	0	0
0	0	1	0	0	0	0	0	0	0	0	1	0
0	0	0	0	0	0	0	0	0	0	0	0	1
0	0	0	0	0	0	0	0	0	0	0	0	0
0	0	0	0	0	0	0	0	1	0	0	1	1
0	0	0	0	0	0	0	0	0	0	0	1	1
0	0	0	0	0	0	0	0	0	0	0	1	0
0	0	0	0	0	0	0	0	0	0	0	0	0
1	0	0	0	0	0	0	0	0	0	0	0	0
0	0	0	1	0	0	1	0	0	1	1	0	1
1	0	0	0	0	0	0	0	1	1	1	1	1
0	0	0	0	0	0	0	0	1	0	1	1	1
0	1	0	0	0	1	1	1	1	1	0	0	0
0	0	0	0	0	0	0	0	1	0	0	1	0
0	0	0	0	0	0	0	0	0	1	0	1	0
0	0	0	0	0	0	0	0	0	1	0	0	0
0	0	0	0	0	0	0	0	0	0	0	1	0
0	0	0	0	0	0	0	0	0	1	0	0	0
0	0	0	0	0	0	0	0	0	0	0	0	0
1	0	0	0	0	0	0	0	0	0	0	1	0
0	0	0	0	0	0	0	0	1	1	0	1	0
0	0	1	0	0	0	0	0	0	0	0	1	0
0	1	0	0	0	0	0	0	0	0	1	1	0
0	0	0	0	0	0	0	0	0	1	1	1	1
0	0	0	0	0	0	0	0	0	1	0	0	1
0	0	0	0	0	0	0	0	0	1	0	0	0
0	0	0	0	0	0	0	0	0	0	0	0	0
0	0	0	0	0	0	0	0	1	0	0	0	0
0	0	0	0	0	0	0	0	0	1	0	0	0
0	0	0	0	0	0	0	0	0	0	0	0	0
0	0	0	0	0	0	0	0	0	0	0	1	0
0	0	1	0	0	0	1	0	0	0	0	0	0
0	0	0	0	1	0	1	0	0	0	0	0	0
0	0	0	0	0	0	0	0	0	0	0	0	0
0	0	0	0	1	0	0	0	0	0	0	0	0
0	0	0	0	0	0	0	0	1	0	0	0	0
0	0	0	0	0	0	0	0	0	0	0	0	0
0	0	0	0	0	0	1	0	1	1	1	1	1
0	0	1	0	1	0	1	0	0	0	0	0	0
0	0	0	0	1	0	1	0	0	1	0	1	0
1	0	0	0	0	0	0	0	0	0	0	1	0
0	0	0	0	0	0	1	0	0	0	0	0	0
